# Role of Oxidative Stress and Antioxidants in Acquired Inner Ear Disorders

**DOI:** 10.3390/antiox11081469

**Published:** 2022-07-27

**Authors:** Megumi Kishimoto-Urata, Shinji Urata, Chisato Fujimoto, Tatsuya Yamasoba

**Affiliations:** Department of Otolaryngology, Graduate School of Medicine, The University of Tokyo, Tokyo 1138655, Japan; kishimoto_meg@yahoo.co.jp (M.K.-U.); surata@m.u-tokyo.ac.jp (S.U.); cfujimoto-tky@umin.ac.jp (C.F.)

**Keywords:** hearing loss, oxidative stress, reactive oxygen species, redox

## Abstract

Oxygen metabolism in the mitochondria is essential for biological activity, and reactive oxygen species (ROS) are produced simultaneously in the cell. Once an imbalance between ROS production and degradation (oxidative stress) occurs, cells are damaged. Sensory organs, especially those for hearing, are constantly exposed during daily life. Therefore, almost all mammalian species are liable to hearing loss depending on their environment. In the auditory pathway, hair cells, spiral ganglion cells, and the stria vascularis, where mitochondria are abundant, are the main targets of ROS. Excessive generation of ROS in auditory sensory organs is widely known to cause sensorineural hearing loss, and mitochondria-targeted antioxidants are candidates for treatment. This review focuses on the relationship between acquired hearing loss and antioxidant use to provide an overview of novel antioxidants, namely medicines, supplemental nutrients, and natural foods, based on clinical, animal, and cultured-cell studies.

## 1. Introduction

Science, technology, and innovation provide us with treatments for diseases, life extension, and diversification of lifestyles. In otology, they have uncovered the mechanisms of sensorineural hearing loss and improved activities of daily living for patients who experience sensorineural hearing loss. Some medications, aging, and living environments with noise exposure cause drug-induced, age-related, and noise-induced hearing loss, respectively. The causes of these acquired hearing losses are fundamentally different. However, cellular damage due to oxidative stress in these pathologies is a common phenomenon at a molecular level. Therefore, understanding the mechanisms of oxidative stress can provide a basis for the treatment of acquired hearing loss.

Eukaryotes have evolved to utilize the energy generated during oxygen metabolism by incorporating mitochondria. In other words, reactive oxygen species (ROS) were inevitably produced in the process of oxygen metabolism. ROS are essential for biological activity and vice versa. The main physiological functions of ROS are of cellular transmitters [1,2] and signaling molecules [3,4]; however, excess generation of ROS is involved in the oxidation of nucleic acids, proteins, carbohydrates, and lipids, resulting in aging and pathological alterations [5]. To prevent cellular toxicity, mitochondria are equipped with systems to detoxify ROS. An imbalance in ROS production and degradation results in mitochondrial dysfunction and the activation of several apoptotic pathways [6]. Oxidative stress in cells is induced by ROS generated by metabolism, ionization by radiation, and carcinogens that act directly on DNA [7]. A portion of oxygen during metabolism is converted to superoxide by one-electron reduction. Two molecules of superoxide are then converted to one molecule of oxygen and one molecule of hydrogen peroxide by superoxide dismutase (SOD), which is a superoxide disproportionating enzyme. Hydrogen peroxide is reduced to water by catalase and glutathione peroxidase [8]. However, the iron-mediated Fenton reaction produces a high level of activated hydroxyl radicals if hydrogen peroxide is not sufficiently reduced. Hydroxyl radicals are also produced by UV irradiation or direct irradiation of water with radiation. Hydroxyl radicals react with the cell membranes to produce lipid peroxides [8].

Cell death in auditory sensory organs is associated with sensorineural hearing loss (SNHL) [9]. In the auditory pathway, hair cells, which contain the sensory epithelium in the cochlea, are the most vulnerable to oxidative stress related to acquired inner ear disorders, such as drug-induced, age-related, and noise-induced hearing loss. Insults to hair cells are irreversible and permanent. Therefore, it is anticipated that mitochondrion-targeted antioxidants will prevent hearing loss before hair cell loss.

The aim of this review was to describe the relationship between acquired hearing loss and antioxidants. Based on clinical, animal, and cultured-cell studies, we provide updated information on novel antioxidants including medicines, supplement nutrients, and natural foods in addition to details about vitamins, especially the relationship between vitamins in neurodegenerative diseases and inner ear disorders. First, we focus on auditory processing and the pathology of hearing loss. Second, we describe the role of oxidative stress in mitochondrial function. Finally, we provide a clear overview of antioxidants used in the treatment of acquired inner ear disorders. 

## 2. Auditory Processing

**Figure 1:**
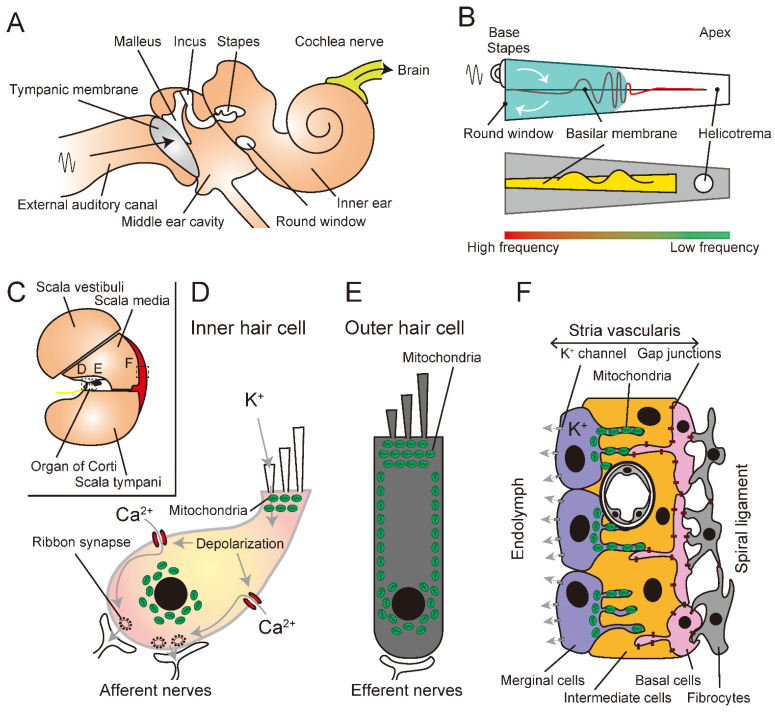
Anatomy of the auditory system. (**A**) Schematic of the auditory system from the external ear to the cochlea nerve. (**B**) Schematic of the theory of traveling sound waves. The sound wave travels from the oval window (stapes) to the round window (white arrows). The sound is attenuated based on the distance from the source, and the sensory epithelium on the basilar membrane is stimulated depending on the sound frequency (red wavy line in the upper panel). High-frequency sounds stimulate the basal portion of the sensory epithelium of the basilar membrane, whereas low-frequency sounds evoke sensory hair cells in the apical portion. (**C**) Anatomy of the inner ear. Sound waves pass through the space filled with endolymph: the scala vestibuli connected to the oval window to the scala tympani to the round window. The organ of Corti is located in the scala media, and the stria vascuralis (red) is part of the lateral wall of the scala media. (**D**–**F**). Representative schematic of the inner ear (**D**), outer hair cells (**E**), and stria vascularis (**F**). Mitochondria are located at the top and around the nucleus in the inner ear (**D**), whereas mitochondria are not only located at the top but are also arranged along the outer wall of the outer hair cells (**E**). Mitochondria in the stria vascularis are located in the fine membranous processes of the marginal cells (**F**).

Sound travels via the outer and middle ears and is encoded in the cochlea of the inner ear (Figure 1A). The function of the cochlea is the conversion of sound into electrical signals based on frequency [10,11]. The sound impinges upon the eardrum, and the vibrations of the eardrum are transmitted through the three small bones of the middle ear, the malleus, incus, and stapes, initiating oscillatory changes in pressure within the coiled cochlea (Figure 1A). Sound vibrations transmitted to the inner ear are transmitted through the perilymph to the scala vestibuli, scala media, and scala tympani, causing the round window to vibrate (Figure 1A–C). This results in vibration of the basilar membrane (Figure 1B). These vibrations are transmitted as waves that travel from the base to the apex of the cochlea [12]. As the traveling wave propagates from the base to the apex of the cochlea, the basal part vibrates [13,14,15]. The vibrations of the basilar membrane decay faster at higher frequencies. Furthermore, the basilar membrane is wider and more flexible at the apex of the cochlea, resulting in a membrane with a lower characteristic frequency. Therefore, the location on the basilar membrane where the amplitude of the traveling wave reaches its maximum value depends on the frequency of the sound; at higher frequencies, it is at the base of the cochlea, and at lower frequencies, it is at the apex (Figure 1B). In other words, the frequency at which the basilar membrane of the cochlea is most likely to vibrate (its characteristic frequency) is determined as a function of the distance from the oval window, and this is the basis of frequency discrimination in hearing. This localization of characteristic frequencies, called tonotopy, is consistent in each part of the auditory conduction pathway in the cortex, up to the auditory cortex [16].

Above the basilar membrane, the organ of Corti is composed of epithelial cells (Figure 1C). The organ of Corti contains one row of inner hair cells that detect most of the afferent information and three rows of outer hair cells that receive projections from efferent nerve fibers (Figure 1D). Vibrations of the basilar membrane open the mechano-electrical transduction channels of the inner hair cells, allowing potassium and calcium ions to flow into the hair cells and generate a receptor current [17]. Depolarization of hair cells by the receptor current activates voltage-gated Ca^2+^ channels, which release the neurotransmitter glutamate from synaptic vesicles, resulting in the transmission of auditory signals to afferent nerve fibers [18]. Conversely, outer hair cells are endowed with the property of dynamic cell length in response to changes in membrane potentials (Figure 1E). It is postulated that this stretching mechanism, which shortens the cell length through depolarization and expands the cell length through hyperpolarization, enhances sensitivity and frequency selectivity of auditory reception through its fast responsivity [19]. In general, outer hair cells are more sensitive to damage than inner hair cells; however, the underlying mechanism has not been fully elucidated. In addition to hair cells, mitochondria are located in the marginal cells of the stria vascularis to maintain a high potassium concentration in the endolymph and to produce endolymphatic potentials (Figure 1F). 

## 3. Sensorineural Hearing Loss

Hearing loss is categorized as conductive, sensorineural, or mixed. Conductive hearing loss is caused by the interruption of sound waves from the external auditory canal to the middle ear cavity (Figure 1A). The cause of SNHL is inner ear dysfunction. Mixed hearing loss is characterized by conductive and sensorineural hearing losses. SNHL is defined as a loss of sensitivity to sound and is broadly classified into two categories: congenital and acquired. Congenital SNHL occurs in approximately 1 in 1000 children and is classified as syndromic when accompanied by symptoms other than hearing loss and as nonsyndromic when only hearing loss is present. Approximately 70% of all nonsyndromic congenital SNHL cases are hereditary [20]. Chromosomal and mitochondrial genetic abnormalities, discussed later, are the main causes of hereditary hearing loss. Drugs, aging, and noise are well-known causes of SNHL.

### 3.1. Drug-Induced Hearing Loss

Drug-induced hearing loss is irreversible and is generally caused by the ingestion of ototoxic drugs. The primary mechanism underlying hearing loss is injury to hair cells [21]. Drugs most frequently associated with ototoxicity in clinical practice are aminoglycoside antibiotics and platinum-based anticancer agents [22,23]. Aminoglycoside cytotoxicity is widespread in the kidney and ear, although the mechanisms are different. Cells in the proximal convoluted tubules of the kidney proliferate so that the changes due to aminoglycosides are reversible [24]. In contrast, cochlear hair cells are unable to regenerate, and ototoxic insults are irreversible [25]. The production of ROS via the apoptotic pathway damages hair cells of the organ of Corti [25]. Basal hair cells that encode high-frequency tones are more vulnerable to injury than apical hair cells that encode low-frequency tones [26]. Gentamicin, an aminoglycoside antimicrobial agent, is known to decrease mitochondrial membrane potential in outer hair cells and cause NADPH production in outer hair cells before cell death [27] and apoptosis of inner hair cells and outer hair cells [28]. These results suggest that gentamicin inhibits mitochondrial metabolism. In addition, aminoglycosides tend to accumulate in the mitochondria within hair cells [29], and the accumulation of ROS associated with mitochondrial dysfunction may cause hearing loss [30]. In fact, gentamicin inhibits protein synthesis in ribosomes within the mitochondria [31] and opens the mitochondrial permeability transition pores [27], resulting in reduced mitochondrial function [32,33]. The A1555G mutation in mitochondrial DNA causes hearing loss because the rRNA conformation is altered and rRNA binds aminoglycosides more strongly than normal [31,34]. In addition to the A1555G mutation, other mitochondrial DNA mutations associated with aminoglycoside hypersensitive hearing loss have recently been reported [35]; however, the mechanism of the interaction between aminoglycosides and rRNA remains unclear [36]. 

Platinum-based anticancer drugs are used to treat squamous cell carcinoma, adenocarcinoma, and undifferentiated carcinomas of the head and neck, lungs, and bladder [37]. Platinum atoms bind to purine bases as DNA cross-linkers, inhibiting cell growth, inactivating the cell cycle, and causing apoptosis of tumor cells [38,39]. However, there are many previous reports on ototoxicity related to cisplatin [40,41]. Cisplatin causes acute phase changes and chronic cytotoxicity in the cochlea. The effects in the acute phase are reversible inhibition of transfer currents and voltage-dependent calcium currents in hair cells [42]. In contrast, long-term toxic reactions elevate ROS levels in the cochlea, leading to cell death by apoptosis [43]. Chronic changes are irreversible and occur in outer hair cells [44,45], stria vascularis [46,47], and spiral ganglion cells [48,49]. Basal hair cells that encode high-frequency tones are more vulnerable to injury than apical hair cells that encode low-frequency tones [50].

The induction of ROS production by cisplatin results in hearing loss. The cochlear sensory epithelium and ROS-producing enzymes NADPH oxidase 3 (NOX3) [51,52] and xanthine oxidase [53] are expressed in spiral ganglion cells. Cisplatin administration elevates ROS levels in the cochlea [54]. In addition, the increased levels of ROS in the cochlea release cytochrome c, an essential factor in the electron transfer system in the mitochondria [41,55]. Increased cytochrome c levels activate the caspase pathway, which induces cell death by apoptosis [56]. It has also been reported that cisplatin treatment upregulates the expression of B-cell/CLL lymphoma 2 (BCL-2)-associated X protein (BAX), an apoptosis-promoting factor, and downregulates the expression of BCL-2, an antiapoptotic protein [57,58].

### 3.2. Age-Related Hearing Loss

Age-related hearing loss is defined as the progressive SNHL associated with aging. Many factors, including genetic, environmental, and medical, have been implicated as causes, although the exact mechanism is unknown [59,60]. Histologically, loss of hair cells, loss of Lassen ganglion cells, and atrophy of the vascular cords have been observed [61,62,63,64]. Mitochondrial function directly affects the pathogenesis of age-related hearing loss. Mitochondrial DNA has an extremely compact structure, unlike nuclear DNA [65]. In other words, once mitochondrial DNA mutates, it mutates at a faster rate than nuclear DNA. In fact, the mutation rate of mitochondrial DNA is more than 10,000 times faster than that of nuclear DNA [66]. Mitochondrial DNA lacks introns [67] and proteins that protect DNA, such as histones. In addition, the mitochondrial DNA repair system is incompletely equipped [68]. These findings suggest that mitochondrial DNA mutations tend to accumulate. Oxidative damage to mitochondrial DNA is known to affect aging [69,70]. Experiments using the senescence-accelerated mouse-prone 8 strain have also shown that aging causes increased oxidative stress, changes in the expression levels of antioxidant enzymes, and activation of the apoptotic pathway [71]. In another animal study, similar results were obtained: SOD is an antioxidant enzyme that detoxifies ROS by degrading superoxide anions.

**Figure 2:**
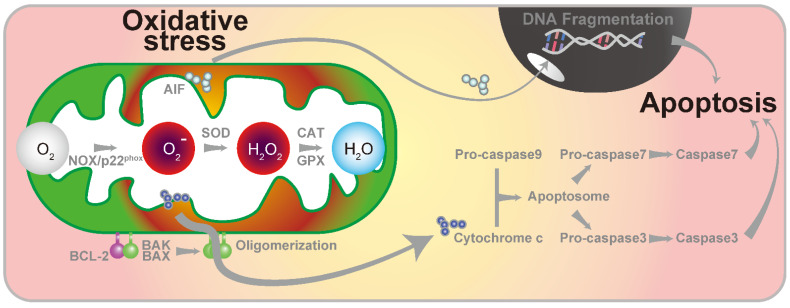
Schematic of cell toxicity due to oxidative stress. AIF, apoptosis-inducing factor; BAK, brassinosteroid insensitive-1-associated receptor kinase; BAX, BCL-2-associated X protein; BCL-2, B-cell/CLL lymphoma 2; CAT, catalase; GPX, glutathione peroxidase; NOX, NADPH oxidase; SOD, superoxide dismutase.

SOD1 knockout mice develop premature age-related hearing loss due to hair cell loss [72]. Mouse models of age-related hearing loss are known to have high rates of mitochondrial DNA mutations and ROS levels [73]. Increases in ROS levels cause a decrease in the mitochondrial membrane potential and apoptosis of hair cells [73]. However, antioxidants can preserve and restore auditory function. Antioxidants, such as vitamin C, vitamin E, and melatonin, improve mitochondrial DNA mutations and show hearing preservation effects [74]. Experiments using C57BL/6 mice, a widely used animal model of age-related hearing loss, have shown that antioxidants are effective in preserving hearing function [75,76]. In contrast, antioxidant treatment increased the antioxidant effect in the inner ear of CBA/J mice but did not improve age-related changes in hair cells or spiral ganglion cells [77]. Apoptotic signaling in age-related hearing loss occurs through mitochondria-dependent endogenous and exogenous pathways, induced by ligands on cell surface receptors. Endogenous apoptosis is regulated by the BCL-2 family of proteins that localize to the mitochondria. Activation of the endogenous pathway indicates that cytochrome c is released from the mitochondrial intermembrane space (Figure 2). The released cytochrome c binds to apoptosis protease-activating factor 1 to form an apoptosome (Figure 2). Apoptosomes activate pro-caspase-9. Active apoptosomes activate caspase-3 and caspase-7 and proceed to the apoptosis pathway (Figure 2). The extrinsic pathway is activated by binding of cell death receptors on the ligand surface, which recruit adapter molecules, such as the fas-associated death domain protein and caspase-8, which in turn recruit caspase-3, and the cleavage and activation of caspase-7 occur, causing apoptosis [78,79,80]. Deletion of the mitochondrial pro-apoptotic gene brassinosteroid insensitive-1-associated receptor kinase prevents age-related hearing loss [76], suggesting that the endogenous pathway of apoptosis is essential for age-related hearing loss.

### 3.3. Noise-Induced Hearing Loss

Noise-induced hearing loss is defined as partially irreversible hearing loss associated with intense sound exposure. The main pathogenesis is mechanical damage [81,82,83], mitochondrial free radical formation [84,85], and apoptosis of hair cells and spiral ganglion cells associated with mitochondrial free radical formation [86]. After noise exposure, mitochondrial free radicals are produced, blood flow in the cochlea is reduced, and eventually, both apoptotic and necrotic cell death of corticotrophs is induced [87]. Apoptosis is associated with the pathogenesis of noise-induced hearing loss because the levels of tumor necrosis factor receptor [88], caspase [89], and apoptosis-inducing factors [90] are elevated. ROS levels in the cochlea increase rapidly after exposure to intense sounds and decrease over time [91]. ROS byproducts, 4-hydroxynonenal and nitrotyrosine, appear 7–10 days after exposure to high-intensity sounds. Immunostaining for prostaglandin-like compounds (8-isoprostanes), a marker of oxidative stress after intense sound exposure, has shown high sensitivity to intense sound in outer hair cells, Lassen ganglion cells, and the stria vascularis [92]. The most important signaling system for determining cell fate, the p38-mitogen-activated protein (MAP) kinase (MAPK)-c-Jun N-terminal kinase (JNK) pathway, is activated by intense sound exposure, inducing cell death in the inner ear [93]. Blocking the MAPK-JNK signaling pathway prevents noise-induced inner ear damage [93], suggesting that ROS and apoptosis are likely involved in the pathogenesis of noise-induced hearing loss. However, it is necessary to consider the pathogenesis of noise-induced hearing loss due to apoptosis, without an increase in ROS levels. Indeed, increased calcium concentration and activation of calcineurin in outer hair cells do not produce ROS but rather induce apoptosis. Vasoactive substances are also induced by intense sound, and inner ear damage associated with reduced local blood flow has also been reported [94,95]. A stable blood supply is essential for cochlear metabolic homeostasis, and reduced cochlear blood flow affects the supply of nutrients and the removal of waste products. Intense sounds are expected to release excessive amounts of glutamate, a neurotransmitter, in the inner hair cells. Based on the above, it cannot be denied that calcium homeostasis, inner ear hemodynamics, and glutamate thyroid excitotoxicity may also be involved in the mechanism behind noise-induced hearing loss [96]. 

## 4. Role of Oxidative Stress

### 4.1. Reactive Oxygen Species

ROS is a general term for substances in which oxygen is transformed into more reactive compounds. The four most common types of radicals are hydroxyl radicals, superoxide anions, hydrogen peroxide, and singlet oxygen. Most of these ROS are produced in the mitochondria [97,98]. ROS occur in intracellular DNA; 1 billion ROS are produced per day. The number of DNA lesions is estimated to be ten thousand per day; however, DNA damage is quickly repaired by intrinsic ROS scavengers [99,100,101]. To remove ROS, antioxidant enzymes such as catalase, SOD, and peroxidase detoxify ROS [102,103,104]. Excess ROS that cannot be degraded by antioxidant enzymes are known to cause various diseases, including neurodegenerative diseases such as Parkinson’s disease [105] and Alzheimer’s disease [106,107], carcinogenesis [108,109], lifestyle-related diseases [110,111], and aging [20,112]. Although ROS are considered toxic products of cellular metabolism, they can function as signaling molecules that regulate many physiological processes [3,113]. ROS play an important role in inducing apoptosis [114,115]. Previous studies indicate that oxidative stress induces apoptosis via both the extrinsic cell death receptor pathway and the intrinsic mitochondrial cell death pathway [116,117].

### 4.2. Mitochondrial Function

Mitochondrial DNA mutations are associated with both syndromic and non-syndromic hearing losses. Syndromic hearing loss is associated with systemic neuromuscular diseases such as mitochondrial disease with stroke (MELAS), mitochondrial encephalomyopathy, chronic progressive exophthalmoplegia, lactic acidosis, and mitochondrial encephalomyopathy with ragged red fibers [118,119,120]. Nonsyndromic hearing loss is maternally inherited. The clinical manifestations are progressive and symmetric, mainly affecting high-frequency bands [121]. Mitochondria were acquired by eukaryotes during evolution and have their own circular DNA. Mitochondrial DNA is composed of 37 genes, and structural abnormalities such as point mutations, deletions, and duplications have been reported. Recently, new mutations were discovered through the development of next-generation sequencing technology [122,123]. DNA is transcribed into mRNA and then translated into proteins via tRNA, which carries amino acids corresponding to three-letter sequences (codons) on the mRNA, and taurine is added to the commentary portion of the codons. In MELAS, this taurine modification is disordered, such that certain codons are not recognized correctly. As a result, proteins cannot be synthesized normally, and mitochondrial function is impaired [124,125,126]. These taurine modifications of mitochondrial tRNA are performed [127] by mitochondrial translation optimization 1 (MTO1) [128] and GTP-binding 3 (GTPBP3) [129]. MTO1 and GTPBP3 are both mitochondrial DNA-encoded rRNA, and the A1555G mutation has been associated with nonsyndromic hearing loss [130,131,132]. MTO1 mutations present symptoms similar to those of MELAS, but the molecular mechanism remains unclear [133]. The A3243G mutation in tRNA, which is related to MELAS, causes SNHL [134,135]. The subunit of mitochondrial oxidative phosphorylation (OXPHOS) enzyme complex I is mitochondrial DNA, and mitochondria with the A3243G mutation have reduced enzyme activity [136]. Reduced OXPHOS activity leads to increased ROS production inducing apoptotic cell death [137]. The mutation rate of mitochondrial DNA is higher in the spiral ganglion than in the organ of Corti, and there is a correlation between the mutation rate and the degree of histological injury, suggesting that OXPHOS activity is related to clinical symptoms as well as activity within organs [138].

## 5. Antioxidant Treatments

Antioxidants are defined as substances that inhibit ROS generation and control oxidative stress. Antioxidants are broadly classified into endogenous antioxidant enzymes produced in vivo and exogenous antioxidants that are supplied from outside the body. Exogenous antioxidants consist of water- and lipid-soluble components. Bipolar antioxidants, such as alpha-lipoic acid, act as antioxidants and restore the antioxidant effects of glutathione, vitamin A, vitamin C, and vitamin E. 

### 5.1. Intrinsic Antioxidants

Endogenous antioxidant enzymes include SOD, glutathione peroxidase (GPX), and catalase (Figure 2). The superoxide anion ROS is neutralized to H_2_O_2_ by SOD. H_2_O_2_ is subsequently decomposed into water and oxygen by GPX and catalase, thereby eliminating ROS. N-acetyl-L-cysteine (NAC), a precursor of glutathione, also exhibits antioxidant properties [139]. Ebselen, a mimetic of GPX, also has antioxidant properties [140]. Excessively produced ROS also activate the transcription factor NF-κβ. As activated NF-κβ is involved in immunity and inflammation and regulates disease onset and exacerbation, antioxidants and n-3 polyunsaturated fatty acids, which contribute to the supplementation and stabilization of radicals, are also used for treatment [141,142,143,144]. Nuclear factor erythroid 2-related factor 2 (NRF2) is a transcriptional activator that plays a protective role against oxidative stress. NRF2 is associated with gentamicin-induced, age-related, and noise-induced hearing loss [145,146]. Activating transcription factor (ATF), a member of the ATF/CREB transcription factor family, was activated in the spiral ganglion and spiral ligament in a noise-induced hearing loss mouse model. Inhibition of ATF3 reduces the expression level of NRF2 and induces ROS accumulation in House Ear Institute-Organ of Corti 1 (HEI-OC1) cells. Nitric oxide (NO) is an important ubiquitous signaling molecule produced by nitric oxide synthase (NOS). NO alters the glucose metabolic pathway from glycolysis to the pentose phosphate pathway and produces reducing equivalents such as NADPH and glutathione (GSH). Endothelial NOS (eNOS)-NO signaling plays an important role in oxidative stress. In HEI-OC1 cells, NO not only protects against H_2_O_2_-induced oxidative stress but also inhibits pyruvate kinase M2 (PKM2). Silencing PKM2 diverts the above-mentioned glucose metabolic pathway [147]. In the auditory pathway, eNOS is expressed in the organ of Corti, stria vascularis, spiral ligament, and spiral ganglion cells in guinea pigs [148]. NADPH oxidases (NOX) produce superoxide free radicals by transferring one electron to oxygen from NADPH (Figure 2). The p22^phox^ protein is a subunit of NOX and is an essential component for the stabilization of NOX. NOX2, 3, and 4 are expressed in the organ of Corti, stria vascularis, and spiral ganglion cells in mice [149]. Sirtuin proteins (SIRT) are a family of signaling proteins that regulate mitochondrial function. SIRT3 is a mitochondrial deacetylase that is expressed in both inner and outer hair cells. SIRT3^−/−^ mice are vulnerable to noise exposure, and in SIRT3^−/−^ mice administered with nicotinamide riboside, an NAD precursor, hearing levels are not protected against noise exposure [149]. 

### 5.2. Extrinsic Antioxidants

#### 5.2.1. Water-Soluble Antioxidants

Known water-soluble antioxidants include methionine, vitamin C, carnitine, riboflavin (vitamin B2), niacin, folic acid, polyphenols, and catechins.

Methionine, an essential amino acid, lowers blood cholesterol levels and removes ROS [140]. Riboflavin and niacin remove lipid peroxides in the presence of GSH [139,150]. Vitamin C acts on hydroxyl radicals and reduces toxicity [151]. Most animals can synthesize their own vitamin C; however, humans and several rodents, such as guinea pigs, are not able to manufacture it themselves because of the absence of the enzyme L-gulonolactone oxidase. Carnitine is a vitamin-like substance that is involved in mitochondrial energy metabolism. Folic acid exerts its antioxidant effect by decreasing blood levels of homocysteine [152]. Polyphenols exert their antioxidant effects by donating hydrogen atoms or electrons to ROS [153]. Ferulic acid reacts with ROS and phenols to exert antioxidant effects. Catechins exert their antioxidant function by inhibiting the oxidation of low-density lipoproteins [153]. 

#### 5.2.2. Lipid-Soluble Antioxidants

Lipid-soluble antioxidants inhibit lipid oxidation reactions in the cell membranes. Β-carotene, vitamin E, astaxanthin, and coenzyme q10 (CoQ10) are widely known and used as supplements. Other potential antioxidants include carotenoids such as α-carotene, γ-carotene, lycopene, and xanthophylls, which are lipid-soluble pigments found in plant collagen. The pro-vitamin A activity of β-carotene removes the singlet oxygen. Vitamin E contributes to the stability of biological membranes. Astaxanthin removes singlet oxygen and is a pro-oxidant; CoQ10 removes lipid radicals in cellular and mitochondrial membranes and inhibits oxidative damage [154]. CoQ10 also reduces oxidized vitamin E radicals after ROS removal [154]. Idebenone is an analog of CoQ10 [155], and Q-TER is a water-soluble CoQ10 analog.

#### 5.2.3. Amphoteric Antioxidants

Antioxidants with both water- and fat-soluble properties include *Ginkgo biloba* and alpha-lipoic acid. *Ginkgo biloba* (EGB761) has antioxidant properties and acts as a scavenger of peroxyl radicals [156]. In fact, in vitro experiments using mouse cochlear neural stem cells revealed that EGB761 regulates apoptosis factors, such as BCL-2, BAX, and Caspase-3, and eventually alleviates hydrogen peroxide-induced oxidative stress [147]. Alpha-lipoic acid not only has its own antioxidant properties but also restores antioxidant capacity by reducing glutathione, vitamin A, vitamin C, and vitamin E, which have lost their antioxidant properties [157]. 

### 5.3. Antioxidants in Acquired Inner Ear Disorders

Oxidative stress plays an important role in SNHL. Accumulation of ROS is considered to be the main cause of acquired hearing loss. The protective effects of antioxidants have been investigated, and it is postulated that antioxidants are effective in many cases of SNHL of an unknown etiology. 

#### 5.3.1. Medicines

##### Drug-Induced Hearing Loss

Edaravone [158], a radical scavenger, was found to improve the auditory brainstem response (ABR) threshold in intraperitoneal (i. p.) gentamicin-treated hearing loss in guinea pigs. NAC was administered for 6 weeks to 53 patients who developed gentamicin-induced hearing loss with hemodialysis [159]. Hearing level on pure tone audiogram (PTA) was improved in the gentamicin-treated group compared to that of the non-treated control group [159]. Ebselen is not a strong antioxidant, although it protects against outer hair cell damage caused by gentamicin ototoxicity. Scanning electron microscopy revealed preservation of the ultrastructure of outer hair cells in ebselen-treated guinea pigs [160]. To confirm the effects of Q-TER on gentamicin ototoxicity, Hartley albino guinea pigs were i. p. administered injection of Q-TER, and the ABR thresholds at 2, 4, 6, 8, 12, 16, and 20 kHz were attenuated by 15 days after treatment. This effect was much clearer in the high-frequency region. Immunohistochemical analysis revealed that the outer hair cell survival rate was also increased compared to that in the sham group [161]. Six guinea pigs were injected i. p. with 200 mg of D-methionine to confirm the effects in vivo on gentamicin ototoxicity. Once-daily administration of D-methionine improved the threshold shift at 3 kHz [162]. L-carnitine, a micronutrient, mitigates gentamicin-induced hearing loss in newborn Guinea pigs by blocking the apoptosis pathway by JNK [163]. L-carnitine preserved not only the ABR thresholds by broadband click stimulation (10 ms duration, 50 µs sample rate) but also the stereocilia structure on the outer hair cells. The efficacy of ebselen has been confirmed in platinum-based drug-induced hearing loss in Wistar rats [164]. The ABR threshold of 49 Wistar rats treated with ebselen for 3 days considerably improved at 4, 8, 16, and 32 kHz, and the ABR threshold shift was within 10 dB [165]. The effects of CoQ10 on cisplatin-induced ototoxicity were confirmed using in vitro and ex vivo experiments with HEI-OC1 cells and rat cochlear explants, respectively. Pretreatment with CoQ10 prevented apoptosis of both inner and outer hair cells in cochlear explants and attenuated ototoxicity via the upregulation of MAPK expression in the HEI-OC1 cells [166]. D-Methionine, an essential amino acid, protects against cisplatin-induced ABR threshold shifts and outer hair cell loss [167]. However, the effect of NAC remains controversial because of the discrepancy in outcomes between mice experiments [168] and human studies [169]. 

##### Age-Related Hearing Loss

Various antioxidants have been investigated for age-related hearing loss, and their protective effects have been confirmed in animal experiments. However, the role of antioxidants in human studies remains controversial. Vitamin C has different effects on humans and animals. CoQ10 significantly prevented age-related changes in both animal experiments and human studies. In fact, administration of alpha lipoic acid and/or CoQ10 inhibits *Bak* expression in the spiral ganglion cells and outer hair cells and prevents Bak-dependent apoptosis in the cochlea of C57BL6/J mice [76]. In a clinical study, 46 patients diagnosed with age-related hearing loss were administered CoQ10 for at least 8 weeks, and the PTA thresholds at 125, 250, 500, 1000, 2000, 4000, and 8000 Hz were improved compared with those at pretreatment [170]. Sixty presbycusis patients were administered Q-TER for 30 days, and the PTA thresholds at 500, 1000, 2000, 4000, and 8000 Hz improved in all patients, and transiently evoked otoacoustic emissions significantly improved [171]. A/J mice are widely used as age-related hearing loss models because of early-onset hearing loss due to hair cell degeneration and reduction in the number of synaptic ribbons. In p22^phox^ deficient A/J mice (nmf333), outer hair cells, synaptic ribbons, and spiral ganglion cells were restored, indicating that NOX inhibitors are candidates for the treatment of age-related hearing loss [149]. Animal studies have reported the effects of NAC at the molecular level, and human studies have shown that folic acid prevents the progression of age-related hearing loss; however, the effects of NAC and folic acid have so far been elusive.

##### Noise-Induced Hearing Loss

GSH is a strong antioxidant that prevents ROS production. Pigmented guinea pigs treated with GSH preserved their hearing after noise exposure (4 kHz octave band noise, 115 dB SPL for 5 h). ABR thresholds at 2, 4, 8, 12, 16, and 20 kHz of GSH-treated animals were improved, and the results were consistent with those of histological examination on cytocochleogram [172,173]. i. p. injected Q-TER protects hearing level against acoustic trauma (6 kHz pure tone noise, 120 dB SPL for 1 h), and ABR thresholds at 2, 4, 8, 12, 16, and 20 kHz gradually improved after noise exposure. Interestingly, the effect of Q-TER at 21 days after noise exposure was similar to that of CoQ10, but the threshold shift 1 h after noise exposure for Q-TER treatment was milder than that for CoQ10 treatment [174]. The effect of D-methionine on noise-induced hearing loss was investigated in adult chinchilla lanigera. They had developed acoustic trauma (4 kHz octave band noise, 105 dB SPL for 6 h), and both ABR thresholds at 2, 4, 6, and 8 kHz and hair cell loss were improved by 21 days after noise exposure [175]. Although the effects of resveratrol [176], a type of polyphenol, ferulic acid [177], which has antioxidant properties similar to those of phenols, and vitamin C [178,179] were confirmed in animal experiments for noise-induced hearing loss, further experiments are necessary to determine their effects. Ebselen mitigates both physiological and pathological changes in noise-induced hearing loss in guinea pigs, and the authors found that the effect is independent of ebselen concentration [180,181], suggesting that the optimal dose of antioxidants is important for treatment. Administration of Annexin, a medical compound of EGB761 and cilostazol, mitigated stereocilia deformation on the outer hair cells and restored the density of efferent cochlear nerve terminals in the root canal after noise exposure in mice [182]. In contrast, NAC, which is effective for age-related hearing loss, has been controversial for the treatment of noise-related hearing loss in both animal experiments [183] and clinical studies [184]. In CBA/J mouse experiments, L-NAME, an inhibitor of eNOS, exacerbated noise-induced hearing loss due to outer hair cell loss, and the number of synaptic ribbons in inner hair cells labeled with CTBP-2 was restored by the administration of L-arginine, a precursor of NO [147]. The NRF2-activating drug 2-cyano-3,12 dioxooleana-1,9 dien-28-imidazole (CDDO-lm) may be a key medication because CDDO-lm protects the hearing level of mice with noise-induced hearing loss by upregulating the expression of genes associated with NRF2, such as *Nqo1, Ho-2, Gclc, Gclm,* and *Txnrd1* [146]. 

#### 5.3.2. Vitamins

In recent years, natural dietetic antioxidants have drawn attention, owing to their potential effects on human health. Large-scale human prospective cohort studies have been performed to confirm the effects of antioxidants for neurodegenerative disorders such as Parkinson’s disease and Alzheimer’s disease.

##### Neurodegenerative Disorders

Parkinson’s disease is distinguished by a significant loss of dopaminergic neurons in the substantia nigra pars compacta as well as the appearance of insoluble protein inclusions known as Lewy bodies. The main pathologies of Alzheimer’s disease are the formation of amyloid-β plaques surrounded by neurons and deposition of hyperphosphorylated tau protein inside the neurons. A meta-analysis of human studies conducted to determine the effects of vitamin C, E, and β-carotene suggested that dietary intake of vitamin E protects against Parkinson’s disease, but not vitamin C and β-carotene intake [185]. Vitamins are not an antioxidant, but it has been widely known as a treatment for Parkinson’s disease. Vitamin D increases dopamine levels and protects dopaminergic neurons against oxidative stress in rats [186]. Low serum vitamin D levels in Parkinson’s disease patients have been reported [186,187]. However, it is still an enigma whether the level of vitamin D is associated with Parkinson’s disease risk. Some reports indicate that scales for symptom severity of Parkinson’s disease (Hoehn and Yahr stage) statistically improved after the administration of vitamin D supplements in a randomized, double-blind, placebo-controlled trial [188]. However, no difference in baseline serum vitamin D levels was observed between high- and low-risk groups according to the Parkinson Associated Risk Syndrome Study [189]. In an animal study including mice with overexpressed human tau proteins, vitamin E intake delayed the development of tau pathology and improved motor function [190]. Additionally, an in vivo study of 341 patients in the Chicago Health and Aging Project revealed that a treatment of higher intake of dietary vitamin E slows the progression of Alzheimer’s disease. Several previous reports indicate the effect of vitamin E on Alzheimer’s disease patients; however, the association between vitamin C and Alzheimer’s disease remains controversial [107,191].

##### Inner Ear Disorder

Antioxidants are expected treatments for SNHL because of analogies of an anatomical structure and pathology between inner ear disorder and neurodegenerative disorders. Dopaminergic neurons, the main target of Parkinson’s disease, innervate the axon terminal of type I spiral ganglion neurons and the axon terminal of the medial olivocochlear nerve [192]. Accumulation of the tau protein, the main cause of Alzheimer’s disease onset, occurs in the stria vascularis by blast exposure [193]. Therefore, vitamins, especially C and E, are potential treatments for SNHL. Indeed, previous reports indicate that vitamins enable improvements in hearing levels among patients with sudden hearing loss [194,195]. In an animal study, vitamin E protected the microstructure of sensory hair cells against cisplatin ototoxicity. Nevertheless, prospective studies reveal that dietary vitamin (A, C, and E) intake is incapable of delaying the progression of age-related hearing loss [196,197,198].

#### 5.3.3. Natural Foods

There are many antioxidants in natural foods, such as fermented papaya [199], fermented milk products [200], extract of hibiscus [201], rosemary essential oil [202], green tea [203], and brown seaweeds [204]. The antioxidant effects of these ingredients have been shown in clinical, animal, and cultured-cell studies. 

Fermentation is a traditional method of food preparation that extends the shelf life and enhances the flavor of food matrices, such as fruits and milk. Recent studies suggest that fermented foods are important components of the human diet owing to their high concentration of health-promoting chemicals, as fermented foods contain a higher fiber content, amino acids, essential fatty acids, vitamins, and minerals [205]. Fermented papaya preparation (FPP) attenuates H_2_O_2_-induced DNA damage and reduces H_2_O_2_-induced p38 phosphorylation, leading to reduced apoptosis. In an in vitro study, FPP reduced amyloid-β precursor protein levels by reducing oxidative stress [206]. The effects of FPP include decreased plasma ROS levels and increased telomere length in the bone marrow [207]. The main components of fermented milk products are proteins and fat; however, these compounds contain antioxidants, such as SOD, catalase, glutathione peroxidase, CoQ10, vitamins (A, C, D_3_, and E), and minerals [200]. In a clinical study, Fardet et al. suggested that dairy milk may have protective effects against cardiovascular diseases and cancer [208]. Hibiscus leaves contain protein, fat, carbohydrate, minerals, vitamins (B_1_, B_2_, and C), and β-carotene [209]. The antioxidant effects of hibiscus leaf extract against free radicals and ROS have been shown by in vivo [210] and in vitro [211] experiments. Systemic antioxidant potential and ascorbic acid and hippuric acid levels in plasma were increased and malondiadehyde urine concentration, which is a biomarker for oxidative stress, was reduced in a randomized, open-label, two-way crossover study [212]. The biological antioxidant properties of rosemary are widely known as free radical terminators and ROS chelators. It is also well-established that polyphenol is the main component of rosemary that produces an antioxidant effect [213]. In vivo and in vitro experiments demonstrate that rosemary extract has the potential as a treatment for Alzheimer’s disease via antioxidant and anti-inflammatory effects, reducing β-amyloid accumulation and regulating acetylcholine activity [214]. Alternatively, memory function was not improved in patients with Alzheimer’s disease who were treated with rosemary extract in a double-blind, randomized, placebo-controlled study [215]. 

However, there are no reports on the relationship between acquired inner ear disorders and antioxidants such as fermented milk products, extract of hibiscus, and rosemary essential oil. In the following section, we will describe the therapeutic potential of green tea and brown seaweed for acquired inner ear disorders.

##### Green Tea

The antioxidant effects of green tea have been extensively investigated. Green tea contains various components with antioxidant activity such as polyphenols, including catechins, minerals (Ca, Mg, Cr, Mn, Fr, Cu, Zn, Mo, Se, Na, P, Co, Sr, Ni, K, F, and Al), and vitamins (B, C, and E). In vitro and animal studies have revealed the effects of polyphenols in green tea against hearing loss. Noise-exposed guinea pigs treated with polyphenol developed hearing loss, but the threshold of ABR and the expression levels of caspase-9 and caspase-3 proteins were low and hair cell loss was significantly decreased compared with those of the non-treated group [216]. Epigallocatechin-3-gallate (EGCG), a polyphenol abundant in green tea, provided protection from adverse events associated with gentamicin-induced ototoxicity. An in vivo and in vitro study revealed that the morphology of hair cells and physiological mechanotransduction currents from hair cells were maintained by regulation of the Notch signaling pathway [217].

##### Brown Seaweeds

Brown seaweed contains polysaccharides, minerals (Ca, Na, P, and K), polyunsaturated fatty acids, and vitamins (A, B_1_, B_12_, C, D, E, riboflavin, niacin, pantothenic acid, and folic acid). Hydrogen peroxide scavenging activity in brown seaweed extracts was higher than that of the commercial oxidants such as vitamin E, butylated hydroxyanisol, and butylated hydroxytoluene in an in vitro study [218]. Dieckol, a pholorotannin polyphenolic compound from brown seaweed, can protect sensory hair cell structure in cochlear explants against gentamicin-induced ototoxicity [219]. 

#### 5.3.4. New Antioxidants

Next, we discuss the novel antioxidants: SIRT3-inhibitor [220], pyrroloquinoline quinone [221], components from the culture broth of *Coprinopsis echinospora* [222], Indian sandalwood oil [223], Twendee-X [224], and vitamin C efflux protein (VCEP) [225]. Whether these new compounds are effective in the treatment of hearing loss remains an enigma. Below, we provide the details including the therapeutic potentials of the compounds. In cultured cochlear cells from C57BL/6 mice, administration of the SIRT3 inhibitor 3-TYP aggravated cochlear damage through the loss of ribbon synapses and hair cells, increase in the apoptosis of hair cells, and ROS production [220]. Pyrroloquinoline quinone (PQQ) is a vitamin expected to act as a redox cofactor. PQQ administration restores mitochondrial respiratory function of H_2_O_2_-treated HEI-OC1 cells. PQQ-treated cells show decreased mitochondrial potential, promoted mitochondrial fusion, and accelerated mitochondrial movement via the SIRT1 pathway [221], suggesting that PQQ is a promising treatment for age-related hearing loss. Ki Dae-Won et al. reported that the extract from *Coprinopsis echinospora* acquired from mushrooms exhibits potent free radical scavenging activity. The authors identified three new compounds: copriqunolinone, spirobenzofuranone, and hydrate of deoxyspirobenzofuran. The antioxidant property of Indian sandalwood oil is stronger than that of vitamin E, as demonstrated by in vitro research on the human keratinocyte cell line and ex vivo studies on human skin explants [226]. A recent clinical study revealed that skin treated with Indian sandalwood oil was protected against blue light exposure [223]. Twendee-X is a relatively new antioxidant containing CoQ10, vitamin C, vitamin B_2_, L-glutamine, L-cystine, crystalline cellulose, magnesium stearate, fumaric acid, succinic acid, micro silicon dioxide, calcium carboxymethyl cellulose, and niacin. Twendee-X decreases phosphorylated tau protein levels in Alzheimer’s disease model mice [227] and attenuates neurotoxicity due to β-amyloid oligomer in a cell line [228]. Furthermore, cognitive function of older participants was improved by Twendee-X intake in a randomized, double-blind, placebo-controlled prospective interventional study. VCEP is a membrane transporter that delivers vitamin C from the blood to the brain through the blood–brain barrier (BBB). The activity of antioxidants in the brain is limited because the BBB prevents the passage of potentially harmful substances. Indeed, almost all the antioxidants except ferulic acid and astaxanthin cannot be transferred to the brain. The BBB is similar to the barrier system in the cochlea. The blood–cochlear barrier is located in the stria vascularis. Therefore, VCEP may play an important role for drug delivery to the cochlea.

#### 5.3.5. Clinical Studies

To understand the effects of antioxidants on SNHL, we searched a database of clinical studies registered at the National Institutes of Health until 3 June 2022. A query-based Clinical Trials search (https://www.clinicaltrials.gov/ accessed on 3 June 2022) was performed to identify the most relevant studies reported in English. The following query was used: ((antioxidant OR antioxidants) AND (hearing loss OR sensorineural hearing loss)). We screened all 34 records and removed 28 studies that were not relevant to the subject matter. Three studies were completed in 2017, 2014, and 2010. All three reports are available on the website, and the last study was only published in print [229]. The first study used β-carotene (18 mg/day), vitamins C (500 mg/day) and E (267 mg/day), and magnesium (315 mg/day) for noise-induced hearing loss. Micronutrients were administered for 4 days until the 4-h music exposure. 

The second used alpha-lipoic acid (1200 mg/day) for cancer patients undergoing treatment with cisplatin, and the administration of alpha-lipoic acid continued until 3 months after the final treatment. The third used NAC (1200 mg/day) for noise-exposed workers, and the supplement was administered for 2 weeks [229]. The threshold shift was not significant in the first study, suggesting that the micronutrients may prevent noise-induced hearing loss. The hearing levels of NAC-treated cancer patients were not significantly different, but the results of pure tone audiometry and distortion-produced optoacoustic emissions were statistically improved in noise-exposed workers.

## 6. Conclusions

Medications, supplemental nutrients, and natural foods have a potential role in the prevention/therapy of hearing loss (Table 1). Ideally, our goal is to prevent the onset of disease via the ingestion of daily nutrients, such as supplements and/or natural foods. The relationship between vitamins and neurodegenerative disorders has been widely confirmed; however, it is practically impossible to completely prevent disease onset and suppress disease progression (see Section 5.3.2 Vitamins). We have addressed the limitations of antioxidant treatments with medications, supplemental nutrients, and natural foods. First, the health benefits of a diet rich in vegetables, fruits, or other antioxidant-rich foods may be due to other substances in the same foods, other diets, or other lifestyles rather than by antioxidants. Second, the dynamics of bioavailability differ among antioxidants. Third, their chemical composition may affect their effectiveness. In fact, vitamin E supplements contain only α-tocopherol, but foods contain eight different chemical forms of vitamin E [230]. Fourth, long-term evaluation of effectiveness of antioxidant treatment is impossible, because dietary intake is dependent on lifestyle. Last, the system of oxidative stress is likely highly complex. Indeed, selenoprotein P impairs thermogenesis in brown adipose tissue by excessive antioxidant effect, suggesting that optimized oxidative stress is necessary to maintain body temperature and that antioxidants have an antagonistic effect [231].

The effects of antioxidants on acquired inner ear disorders are poorly understood because almost all the reports discussed here are animal studies. Further clinical research is required to clarify the mechanism underlying acquired inner ear disorders and the effects of antioxidants on hearing loss.

## Figures and Tables

**Table 1:** Summary of antioxidants for their potential role in the prevention/therapy of hearing loss.

Antioxidants		Potential Role in the Prevention/Therapy of Hearing Loss (References)
Medicine	Edaravone	Preserved ABR waves against ototoxic drugs in an animal experiment [158].
	NAC	Protected hair cell strucure against cisplatin-induced ototoxicity in animal experiments [168,183], and ABR threshold was preserved against drug- and noise-induced hearing loss in human studies [159,184,229].
	Ebselen	Preserved hair cells and ABR threshold against drug- and noise-induced hearing loss in animal experiments [160,164,165,180,181].
	Q-ter	Has a potential to preserve hearing level against noise-induced hearing loss in a clnical study [171] and ameliorated ABR threshold after ototoxic drug and noise exposure [161,174].
	Methionine	The hair cell structure and ABR threshold were preserved against drug- and noise-induced hearing loss in animal experiments [162,167,175].
	L-carnitine	Prevented changes in hearing threshold and cochlear damage in newborn guinea pigs exposed to gentamicin in utero [163]
	CoQ10	Prevented ototoxic apoptosis of the hair cells in a culture experiment [166], and PTA threshold was improved in a human study [170].
	Vitamin C	ABR threshold was preserved against ototoxic drugs in an animal experiment [179].
	GSH	Improved ABR threshold shift after noise exposure, and sensory epithelium was preserved in animal experiments [172,173].
	Resveratrol	Prevented ABR threshold shift after noise exposure in an animal experiment [176].
	Anenexin	Protected stereocillia, hair cells, and cochlear nerve ater noise exposure in an animal experiment [182].
Natural foods	Green tea	Hair cell loss against noise exposure was decreased (216), and mechanotransduction currents from hair cells against ototoxic drugs were maintained [217].
	Brown seaweeds	Enabled to prevent cell damage due to ototoxic drugs in culture experiments [218,219]
New antioxidants	SIRT3-inhibitor	Aggravates cochlear damage due to loss of ribbon synapses and hair cells, increase in apoptosis of hair cells, and ROS production [220].
	Pyrroloquinoline quinone	Pyrroloquinoline quinone-treated cells showed decreased mitochondrial potential, promoted mitochondrial fusion, and accelerated mitochondrial movement [221].

NAC, N-acetyl-L-cysteine; CoQ10, coenzyme q10; GSH, glutathione; SIRT, Sirtuin proteins; CoQ, coenzyme q10, ABR, auditory brainstem response, PTA: pure tone audiogram; ROS, reactive oxygen species.

## References

[1] Beckhauser T.F., Francis-Oliveira J., De Pasquale R. (2016). Reactive Oxygen Species: Physiological and Physiopathological Effects on Synaptic Plasticity. J. Exp. Neurosci..

[2] Beltrán González A.N., López Pazos M.I., Calvo D.J. (2020). Reactive Oxygen Species in the Regulation of the GABA Mediated Inhibitory Neurotransmission. Neuroscience.

[3] Sena L.A., Chandel N.S. (2012). Physiological roles of mitochondrial reactive oxygen species. Mol. Cell.

[4] Zhang J., Wang X., Vikash V., Ye Q., Wu D., Liu Y., Dong W. (2016). ROS and ROS-mediated cellular signaling. Oxid. Med. Cell. Longev..

[5] Marchi S., Giorgi C., Suski J.M., Agnoletto C., Bononi A., Bonora M., De Marchi E., Missiroli S., Patergnani S., Poletti F. (2012). Mitochondria-ros crosstalk in the control of cell death and aging. J. Signal Transduct..

[6] Simon H.-U., Haj-Yehia A., Levi-Schaffer F. (2000). Role of reactive oxygen species (ROS) in apoptosis induction. Apoptosis.

[7] Chompoosor A., Saha K., Ghosh P.S., Macarthy D.J., Miranda O.R., Zhu Z., Arcaro K.F., Rotello V.M. (2010). The role of surface functionality on acute cytotoxicity, ROS generation and DNA damage by cationic gold nanoparticles. Small.

[8] Michalska P., León R. (2020). When it comes to an end: Oxidative stress crosstalk with protein aggregation and neuroinflammation induce neurodegeneration. Antioxidants.

[9] Wu J., Ye J., Kong W., Zhang S., Zheng Y. (2020). Programmed cell death pathways in hearing loss: A review of apoptosis, autophagy and programmed necrosis. Cell Prolif..

[10] Fettiplace R. (2020). Diverse Mechanisms of Sound Frequency Discrimination in the Vertebrate Cochlea. Trends Neurosci..

[11] von Békésy G., Peake W.T. (1990). Experiments in hearing. J. Acoust. Soc. Am..

[12] Hudspeth A.J. (2014). Integrating the active process of hair cells with cochlear function. Nat. Rev. Neurosci..

[13] Davis H. (1958). Transmission and transduction in the cochlea. Laryngoscope.

[14] Ren T. (2002). Longitudinal pattern of basilar membrane vibration in the sensitive cochlea. Proc. Natl. Acad. Sci. USA.

[15] Lee H.Y., Raphael P.D., Park J., Ellerbee A.K., Applegate B.E., Oghalai J.S. (2015). Noninvasive in vivo imaging reveals differences between tectorial membrane and basilar membrane traveling waves in the mouse cochlea. Proc. Natl. Acad. Sci. USA.

[16] Tsukano H., Horie M., Ohga S., Takahashi K., Kubota Y., Hishida R., Takebayashi H., Shibuki K. (2017). Reconsidering Tonotopic Maps in the Auditory Cortex and Lemniscal Auditory Thalamus in Mice. Front. Neural Circuits.

[17] Xiong W., Grillet N., Elledge H.M., Wagner T.F.J., Zhao B., Johnson K.R., Kazmierczak P., Müller U. (2012). TMHS Is an Integral Component of the Mechanotransduction Machinery of Cochlear Hair Cells. Cell.

[18] Wang H.C., Lin C.-C., Cheung R., Zhang-Hooks Y., Agarwal A., Ellis-Davies G., Rock J., Bergles D.E. (2015). Spontaneous Activity of Cochlear Hair Cells Triggered by Fluid Secretion Mechanism in Adjacent Support Cells. Cell.

[19] Ashmore J. (2008). Cochlear outer hair cell motility. Physiol. Rev..

[20] Blagosklonny M. (2008). V Aging: Ros or tor. Cell Cycle.

[21] Huth M.E., Ricci A.J., Cheng A. (2011). Mechanisms of aminoglycoside ototoxicity and targets of hair cell protection. Int. J. Otolaryngol..

[22] Kroese A.B.A., van den Bercken J. (1980). Dual action of ototoxic antibiotics on sensory hair cells. Nature.

[23] Schacht J., Talaska A.E., Rybak L.P. (2012). Cisplatin and aminoglycoside antibiotics: Hearing loss and its prevention. Anat. Rec. Adv. Integr. Anat. Evol. Biol..

[24] Duffield J.S., Bonventre J. (2005). V Kidney tubular epithelium is restored without replacement with bone marrow–derived cells during repair after ischemic injury. Kidney Int..

[25] Cheng A.G., Cunningham L.L., Rubel E.W. (2003). Hair cell death in the avian basilar papilla: Characterization of the in vitro model and caspase activation. J. Assoc. Res. Otolaryngol..

[26] Jensen-Smith H.C., Hallworth R., Nichols M.G. (2012). Gentamicin rapidly inhibits mitochondrial metabolism in high-frequency cochlear outer hair cells. PLoS ONE.

[27] Dehne N., Rauen U., de Groot H., Lautermann J. (2002). Involvement of the mitochondrial permeability transition in gentamicin ototoxicity. Hear. Res..

[28] Suzuki M., Ushio M., Yamasoba T. (2008). Time course of apoptotic cell death in guinea pig cochlea following intratympanic gentamicin application. Acta Otolaryngol..

[29] Marcotti W., Van Netten S.M., Kros C.J. (2005). The aminoglycoside antibiotic dihydrostreptomycin rapidly enters mouse outer hair cells through the mechano-electrical transducer channels. J. Physiol..

[30] Abi-Hachem R.N., Zine A., Van De Water T.R. (2010). The injured cochlea as a target for inflammatory processes, initiation of cell death pathways and application of related otoprotective strategies. Recent Patents CNS Drug Discov..

[31] Hobbie S.N., Akshay S., Kalapala S.K., Bruell C.M., Shcherbakov D., Böttger E.C. (2008). Genetic analysis of interactions with eukaryotic rRNA identify the mitoribosome as target in aminoglycoside ototoxicity. Proc. Natl. Acad. Sci. USA.

[32] Halestrap A.P., Clarke S.J., Javadov S.A. (2004). Mitochondrial permeability transition pore opening during myocardial reperfusion—a target for cardioprotection. Cardiovasc. Res..

[33] Halestrap A.P. (2009). What is the mitochondrial permeability transition pore?. J. Mol. Cell. Cardiol..

[34] Tono T., Kiyomizu K., Matsuda K., Komune S., Usami S., Abe S., Shinkawa H. (2001). Different clinical characteristics of aminoglycoside-induced profound deafness with and without the 1555 A-->G mitochondrial mutation. ORL J. Otorhinolaryngol. Relat. Spec..

[35] Dowlati M.A., Derakhshandeh-Peykar P., Houshmand M., Farhadi M., Shojaei A., Fallah M., Mohammadi E., Tajdini A., Arastoo S., Tavakkoly-Bazzaz J. (2013). Novel nucleotide changes in mutational analysis of mitochondrial 12SrRNA gene in patients with nonsyndromic and aminoglycoside-induced hearing loss. Mol. Biol. Rep..

[36] Böttger E.C. (2010). Mutant A1555G mitochondrial 12S rRNA and aminoglycoside susceptibility. Antimicrob. Agents Chemother..

[37] Vokes E.E. (2010). Induction chemotherapy for head and neck cancer: Recent data. Oncologist.

[38] Damsma G.E., Alt A., Brueckner F., Carell T., Cramer P. (2007). Mechanism of transcriptional stalling at cisplatin-damaged DNA. Nat. Struct. Mol. Biol..

[39] Chu G. (1994). Cellular responses to cisplatin. The roles of DNA-binding proteins and DNA repair. J. Biol. Chem..

[40] Rybak L.P., Ramkumar V. (2007). Ototoxicity. Kidney Int..

[41] Rybak L.P., Whitworth C.A., Mukherjea D., Ramkumar V. (2007). Mechanisms of cisplatin-induced ototoxicity and prevention. Hear. Res..

[42] Kimitsuki T., Nakagawa T., Hisashi K., Komune S., Komiyama S. (1993). Cisplatin blocks mechano-electric transducer current in chick cochlear hair cells. Hear. Res..

[43] García-Berrocal J.R., Nevado J., Ramírez-Camacho R., Sanz R., González-García J.A., Sánchez-Rodríguez C., Cantos B., España P., Verdaguer J.M., Trinidad Cabezas A. (2007). The anticancer drug cisplatin induces an intrinsic apoptotic pathway inside the inner ear. Br. J. Pharmacol..

[44] Kaltenbach J.A., Rachel J.D., Mathog T.A., Zhang J., Falzarano P.R., Lewandowski M. (2002). Cisplatin-induced hyperactivity in the dorsal cochlear nucleus and its relation to outer hair cell loss: Relevance to tinnitus. J. Neurophysiol..

[45] Alam S.A., Ikeda K., Oshima T., Suzuki M., Kawase T., Kikuchi T., Takasaka T. (2000). Cisplatin-induced apoptotic cell death in Mongolian gerbil cochlea. Hear. Res..

[46] Lee J.E., Nakagawa T., Kita T., Kim T.S., Iguchi F., Endo T., Shiga A., Lee S.H., Ito J. (2004). Mechanisms of apoptosis induced by cisplatin in marginal cells in mouse stria vascularis. ORL J. Otorhinolaryngol. Relat. Spec..

[47] Meech R.P., Campbell K.C.M., Hughes L.P., Rybak L.P. (1998). A semiquantitative analysis of the effects of cisplatin on the rat stria vascularis. Hear. Res..

[48] Lee J.E., Nakagawa T., Kim T.S., Iguchi F., Endo T., Dong Y., Yuki K., Naito Y., Lee S.H., Ito J. (2003). A novel model for rapid induction of apoptosis in spiral ganglions of mice. Laryngoscope.

[49] Bowers W.J., Chen X., Guo H., Frisina D.R., Federoff H.J., Frisina R.D. (2002). Neurotrophin-3 transduction attenuates cisplatin spiral ganglion neuron ototoxicity in the cochlea. Mol. Ther..

[50] Anniko M., Sobin A. (1986). Cisplatin: Evaluation of its ototoxic potential. Am. J. Otolaryngol..

[51] Bánfi B., Malgrange B., Knisz J., Steger K., Dubois-Dauphin M., Krause K.-H. (2004). NOX3, a superoxide-generating NADPH oxidase of the inner ear. J. Biol. Chem..

[52] Mohri H., Ninoyu Y., Sakaguchi H., Hirano S., Saito N., Ueyama T. (2021). Nox3-derived superoxide in cochleae induces sensorineural hearing loss. J. Neurosci..

[53] Ikeda K., Sunose H., Takasaka T. (1993). Effects of free radicals on the intracellular calcium concentration in the isolated outer hair cell of the guinea pig cochlea. Acta Otolaryngol..

[54] Kopke R.D., Liu W., Gabaizadeh R., Jacono A., Feghali J. (1997). Use of Organotypic Cultures of Corti’s Organ to. Ann. J. Otol..

[55] Casares C., Ramírez-Camacho R., Trinidad A., Roldán A., Jorge E., García-Berrocal J.R. (2012). Reactive oxygen species in apoptosis induced by cisplatin: Review of physiopathological mechanisms in animal models. Eur. Arch. Oto-Rhino-Laryngol..

[56] Watanabe K., Inai S., Jinnouchi K., Baba S., Yagi T. (2003). Expression of caspase-activated deoxyribonuclease (CAD) and caspase 3 (CPP32) in the cochlea of cisplatin (CDDP)-treated guinea pigs. Auris. Nasus. Larynx.

[57] Sheth S., Mukherjea D., Rybak L.P., Ramkumar V. (2017). Mechanisms of cisplatin-induced ototoxicity and otoprotection. Front. Cell. Neurosci..

[58] Rybak L.P., Mukherjea D., Jajoo S., Ramkumar V. (2009). Cisplatin ototoxicity and protection: Clinical and experimental studies. Tohoku J. Exp. Med..

[59] Yamasoba T., Lin F.R., Someya S., Kashio A., Sakamoto T., Kondo K. (2013). Current concepts in age-related hearing loss: Epidemiology and mechanistic pathways. Hear. Res..

[60] Cruickshanks K.J., Zhan W., Zhong W. (2010). Epidemiology of age-related hearing impairment. The Aging Auditory System.

[61] Lee K.-Y. (2013). Pathophysiology of age-related hearing loss (peripheral and central). Korean J. Audiol..

[62] Gates G.A., Mills J.H. (2005). Presbycusis. Lancet.

[63] Schuknecht H.F. (1955). Presbycusis. Laryngoscope.

[64] Schuknecht H.F., Gacek M.R. (1993). Cochlear pathology in presbycusis. Ann. Otol. Rhinol. Laryngol..

[65] Grivell L.A. (1989). Mitochondrial DNA. Small, beautiful and essential. Nature.

[66] Linnane A.W., Marzuki S., Ozawa T., Tanaka M. (1989). Mitochondrial DNA mutations as an important contributor to ageing and degenerative diseases. Lancet.

[67] Anderson S., Bankier A.T., Barrell B.G., de Bruijn M.H.L., Coulson A.R., Drouin J., Eperon I.C., Nierlich D.P., Roe B.A., Sanger F. (1981). Sequence and organization of the human mitochondrial genome. Nature.

[68] Clayton D.A., Doda J.N., Friedberg E.C. (1974). The absence of a pyrimidine dimer repair mechanism in mammalian mitochondria. Proc. Natl. Acad. Sci. USA.

[69] Bai U., Seidman M.D., Hinojosa R., Quirk W.S. (1997). Mitochondrial DNA deletions associated with aging and possibly presbycusis: A human archival temporal bone study. Am. J. Otol..

[70] Ozawa T. (1995). Mechanism of somatic mitochondrial DNA mutations associated with age and diseases. Biochim. Biophys. Acta (BBA)-Molecular Basis Dis..

[71] Menardo J., Tang Y., Ladrech S., Lenoir M., Casas F., Michel C., Bourien J., Ruel J., Rebillard G., Maurice T. (2012). Oxidative stress, inflammation, and autophagic stress as the key mechanisms of premature age-related hearing loss in SAMP8 mouse Cochlea. Antioxid. Redox Signal..

[72] McFadden S.L., Ding D., Reaume A.G., Flood D.G., Salvi R.J. (1999). Age-related cochlear hair cell loss is enhanced in mice lacking copper/zinc superoxide dismutase. Neurobiol. Aging.

[73] Darrat I., Ahmad N., Seidman K., Seidman M.D. (2007). Auditory research involving antioxidants. Curr. Opin. Otolaryngol. Head Neck Surg..

[74] Seidman M.D. (2000). Effects of dietary restriction and antioxidants on presbyacusis. Laryngoscope.

[75] Heman-Ackah S.E., Juhn S.K., Huang T.C., Wiedmann T.S. (2010). A combination antioxidant therapy prevents age-related hearing loss in C57BL/6 mice. Otolaryngol. neck Surg. Off. J. Am. Acad. Otolaryngol. Neck Surg..

[76] Someya S., Xu J., Kondo K., Ding D., Salvi R.J., Yamasoba T., Rabinovitch P.S., Weindruch R., Leeuwenburgh C., Tanokura M. (2009). Age-related hearing loss in C57BL/6J mice is mediated by Bak-dependent mitochondrial apoptosis. Proc. Natl. Acad. Sci. USA.

[77] Sha S.-H., Kanicki A., Halsey K., Wearne K.A., Schacht J. (2012). Antioxidant-enriched diet does not delay the progression of age-related hearing loss. Neurobiol. Aging.

[78] Cohen G.M. (1997). Caspases: The executioners of apoptosis. Biochem. J..

[79] Lindsten T., Ross A.J., King A., Zong W.-X., Rathmell J.C., Shiels H.A., Ulrich E., Waymire K.G., Mahar P., Frauwirth K. (2000). The combined functions of proapoptotic Bcl-2 family members bak and bax are essential for normal development of multiple tissues. Mol. Cell.

[80] Youle R.J., Strasser A. (2008). The BCL-2 protein family: Opposing activities that mediate cell death. Nat. Rev. Mol. Cell Biol..

[81] Spoendlin H. (1971). Primary structural changes in the organ of Corti after acoustic overstimulation. Acta Otolaryngol..

[82] Mulroy M.J., Henry W.R., McNeil P.L. (1998). Noise-induced transient microlesions in the cell membranes of auditory hair cells. Hear. Res..

[83] Slepecky N. (1986). Overview of mechanical damage to the inner ear: Noise as a tool to probe cochlear function. Hear. Res..

[84] Lim D.J., Melnick W. (1971). Acoustic damage of the cochlea: A scanning and transmission electron microscopic observation. Arch. Otolaryngol..

[85] Yamane H., Nakai Y., Takayama M., Iguchi H., Nakagawa T., Kojima A. (1995). Appearance of free radicals in the guinea pig inner ear after noise-induced acoustic trauma. Eur. Arch. Oto-Rhino-Laryngol..

[86] Hong O., Kerr M.J., Poling G.L., Dhar S. (2013). Understanding and preventing noise-induced hearing loss. Dis Mon.

[87] Henderson D., Bielefeld E.C., Harris K.C., Hu B.H. (2006). The role of oxidative stress in noise-induced hearing loss. Ear Hear..

[88] Hu B.H., Cai Q., Manohar S., Jiang H., Ding D., Coling D.E., Zheng G., Salvi R. (2009). Differential expression of apoptosis-related genes in the cochlea of noise-exposed rats. Neuroscience.

[89] Nicotera T.M., Hu B.H., Henderson D. (2003). The caspase pathway in noise-induced apoptosis of the chinchilla cochlea. J. Assoc. Res. Otolaryngol..

[90] Yamashita D., Miller J.M., Jiang H.-Y., Minami S.B., Schacht J. (2004). AIF and EndoG in noise-induced hearing loss. Neuroreport.

[91] Ohlemiller K.K., Wright J.S., Dugan L.L. (1999). Early elevation of cochlear reactive oxygen species following noise exposure. Audiol. Neurootol..

[92] Ohinata Y., Miller J.M., Altschuler R.A., Schacht J. (2000). Intense noise induces formation of vasoactive lipid peroxidation products in the cochlea. Brain Res..

[93] Pirvola U., Xing-Qun L., Virkkala J., Saarma M., Murakata C., Camoratto A.M., Walton K.M., Ylikoski J. (2000). Rescue of hearing, auditory hair cells, and neurons by CEP-1347/KT7515, an inhibitor of c-Jun N-terminal kinase activation. J. Neurosci..

[94] Perlman H.B., Kimura R. (1962). Cochlear blood flow in acoustic trauma. Acta Otolaryngol..

[95] Hawkins Jr J.E. (1971). The role of vasoconstriction in noise-induced hearing loss. Ann. Otol. Rhinol. Laryngol..

[96] Puel J.L., Ruel J., Gervais d’Aldin C., Pujol R. (1998). Excitotoxicity and repair of cochlear synapses after noise-trauma induced hearing loss. Neuroreport.

[97] Turrens J.F. (2003). Mitochondrial formation of reactive oxygen species. J. Physiol..

[98] Balaban R.S., Nemoto S., Finkel T. (2005). Mitochondria, oxidants, and aging. Cell.

[99] Lindahl T., Wood R.D. (1999). Quality control by DNA repair. Science.

[100] Hemnani T., Parihar M.S. (1998). Reactive oxygen species and oxidative DNA damage. Indian J. Physiol. Pharmacol..

[101] Martinet W., Knaapen M.W.M., De Meyer G.R.Y., Herman A.G., Kockx M.M. (2002). Elevated levels of oxidative DNA damage and DNA repair enzymes in human atherosclerotic plaques. Circulation.

[102] Sies H. (1997). Oxidative stress: Oxidants and antioxidants. Exp. Physiol..

[103] Vertuani S., Angusti A., Manfredini S. (2004). The Antioxidants and Pro-Antioxidants Network: An Overview. Curr. Pharm. Des..

[104] Orr W.C., Sohal R.S. (1994). Extension of life-span by overexpression of superoxide dismutase and catalase in Drosophila melanogaster. Science.

[105] Jing X., Shi H., Zhang C., Ren M., Han M., Wei X., Zhang X., Lou H. (2015). Dimethyl fumarate attenuates 6-OHDA-induced neurotoxicity in SH-SY5Y cells and in animal model of Parkinson’s disease by enhancing Nrf2 activity. Neuroscience.

[106] Sano M., Ernesto C., Thomas R.G., Klauber M.R., Schafer K., Grundman M., Woodbury P., Growdon J., Cotman C.W., Pfeiffer E. (1997). A controlled trial of selegiline, alpha-tocopherol, or both as treatment for Alzheimer’s disease. N. Engl. J. Med..

[107] Engelhart M.J., Geerlings M.I., Ruitenberg A., van Swieten J.C., Hofman A., Witteman J.C.M., Breteler M.M.B. (2002). Dietary intake of antioxidants and risk of Alzheimer disease. JAMA.

[108] Pelicano H., Carney D., Huang P. (2004). ROS stress in cancer cells and therapeutic implications. Drug Resist. Updat..

[109] Moloney J.N., Cotter T.G. (2018). ROS signalling in the biology of cancer. Semin. Cell Dev. Biol..

[110] Wu D., Cederbaum A.I. (2003). Alcohol, oxidative stress, and free radical damage. Alcohol Res. Health.

[111] Radak Z., Zhao Z., Koltai E., Ohno H., Atalay M. (2013). Oxygen consumption and usage during physical exercise: The balance between oxidative stress and ROS-dependent adaptive signaling. Antioxid. Redox Signal..

[112] Davalli P., Mitic T., Caporali A., Lauriola A., D’Arca D. (2016). ROS, cell senescence, and novel molecular mechanisms in aging and age-related diseases. Oxid. Med. Cell. Longev..

[113] Thannickal V.J., Fanburg B.L. (2000). Reactive oxygen species in cell signaling. Am. J. Physiol. Cell. Mol. Physiol..

[114] Volpe C.M.O., Villar-Delfino P.H., Dos Anjos P.M.F., Nogueira-Machado J.A. (2018). Cellular death, reactive oxygen species (ROS) and diabetic complications. Cell Death Dis..

[115] Su L.-J., Zhang J.-H., Gomez H., Murugan R., Hong X., Xu D., Jiang F., Peng Z.-Y. (2019). Reactive oxygen species-induced lipid peroxidation in apoptosis, autophagy, and ferroptosis. Oxid. Med. Cell. Longev..

[116] Sinha K., Das J., Pal P.B., Sil P.C. (2013). Oxidative stress: The mitochondria-dependent and mitochondria-independent pathways of apoptosis. Arch. Toxicol..

[117] Nagai H., Noguchi T., Homma K., Katagiri K., Takeda K., Matsuzawa A., Ichijo H. (2009). Ubiquitin-like Sequence in ASK1 Plays Critical Roles in the Recognition and Stabilization by USP9X and Oxidative Stress-Induced Cell Death. Mol. Cell.

[118] Schon E.A., Bonilla E., DiMauro S. (1997). Mitochondrial DNA mutations and pathogenesis. J. Bioenerg. Biomembr..

[119] Chomyn A. (1998). The myoclonic epilepsy and ragged-red fiber mutation provides new insights into human mitochondrial function and genetics. Am. J. Hum. Genet..

[120] Sue C.M., Lipsett L.J., Crimmins D.S., Tsang C.S., Boyages S.C., Presgrave C.M., Gibson W.P., Byrne E., Morris J.G. (1998). Cochlear origin of hearing loss in MELAS syndrome. Ann. Neurol..

[121] Kokotas H., Petersen M.B., Willems P.J. (2007). Mitochondrial deafness. Clin. Genet..

[122] Carroll C.J., Brilhante V., Suomalainen A. (2014). Next-generation sequencing for mitochondrial disorders. Br. J. Pharmacol..

[123] Ohtake A., Murayama K., Mori M., Harashima H., Yamazaki T., Tamaru S., Yamashita Y., Kishita Y., Nakachi Y., Kohda M. (2014). Diagnosis and molecular basis of mitochondrial respiratory chain disorders: Exome sequencing for disease gene identification. Biochim. Biophys. Acta.

[124] Tsutomu S., Asuteka N., Takeo S. (2011). Human mitochondrial diseases caused by lack of taurine modification in mitochondrial tRNAs. Wiley Interdiscip. Rev. RNA.

[125] Kirino Y., Yasukawa T., Ohta S., Akira S., Ishihara K., Watanabe K., Suzuki T. (2004). Codon-specific translational defect caused by a wobble modification deficiency in mutant tRNA from a human mitochondrial disease. Proc. Natl. Acad. Sci. USA.

[126] Morscher R.J., Ducker G.S., Li S.H.-J., Mayer J.A., Gitai Z., Sperl W., Rabinowitz J.D. (2018). Mitochondrial translation requires folate-dependent tRNA methylation. Nature.

[127] Umeda N., Suzuki T., Yukawa M., Ohya Y., Shindo H., Watanabe K., Suzuki T. (2005). Mitochondria-specific RNA-modifying enzymes responsible for the biosynthesis of the wobble base in mitochondrial tRNAs: Implications for the molecular pathogenesis of human mitochondrial diseases. J. Biol. Chem..

[128] Li X., Guan M.-X. (2002). A human mitochondrial GTP binding protein related to tRNA modification may modulate phenotypic expression of the deafness-associated mitochondrial 12S rRNA mutation. Mol. Cell. Biol..

[129] Villarroya M., Prado S., Esteve J.M., Soriano M.A., Aguado C., Pérez-Martínez D., Martínez-Ferrandis J.I., Yim L., Victor V.M., Cebolla E. (2008). Characterization of human GTPBP3, a GTP-binding protein involved in mitochondrial tRNA modification. Mol. Cell. Biol..

[130] Bykhovskaya Y., Mengesha E., Wang D., Yang H., Estivill X., Shohat M., Fischel-Ghodsian N. (2004). Phenotype of non-syndromic deafness associated with the mitochondrial A1555G mutation is modulated by mitochondrial RNA modifying enzymes MTO1 and GTPBP3. Mol. Genet. Metab..

[131] Inoue K., Takai D., Soejima A., Isobe K., Yamasoba T., Oka Y., Goto Y., Hayashi J. (1996). Mutant mtDNA at 1555 A to G in 12S rRNA gene and hypersusceptibility of mitochondrial translation to streptomycin can be co-transferred to rho 0 HeLa cells. Biochem. Biophys. Res. Commun..

[132] Yamasoba T., Goto Y.I., Oka Y., Nishino I., Tsukuda K., Nonaka I. (2002). Atypical muscle pathology and a survey of cis-mutations in deaf patients harboring a 1555 A-to-G point mutation in the mitochondrial ribosomal RNA gene. Neuromuscul. Disord..

[133] Ghezzi D., Baruffini E., Haack T.B., Invernizzi F., Melchionda L., Dallabona C., Strom T.M., Parini R., Burlina A.B., Meitinger T. (2012). Mutations of the mitochondrial-tRNA modifier MTO1 cause hypertrophic cardiomyopathy and lactic acidosis. Am. J. Hum. Genet..

[134] Nagata H., Kumahara K., Tomemori T., Arimoto Y., Isoyama K., Yoshida K., Konno A. (2001). Frequency and clinical features of patients with sensorineural hearing loss associated with the A3243G mutation of the mitochondrial DNA in otorhinolaryngic clinics. J. Hum. Genet..

[135] Kadowaki T., Kadowaki H., Mori Y., Tobe K., Sakuta R., Suzuki Y., Tanabe Y., Sakura H., Awata T., Goto Y. (1994). A subtype of diabetes mellitus associated with a mutation of mitochondrial DNA. N. Engl. J. Med..

[136] Yasukawa T., Kirino Y., Ishii N., Holt I.J., Jacobs H.T., Makifuchi T., Fukuhara N., Ohta S., Suzuki T., Watanabe K. (2005). Wobble modification deficiency in mutant tRNAs in patients with mitochondrial diseases. FEBS Lett..

[137] Zapico S.C., Ubelaker D.H. (2013). mtDNA mutations and their role in aging, diseases and forensic sciences. Aging Dis..

[138] Takahashi K., Merchant S.N., Miyazawa T., Yamaguchi T., McKenna M.J., Kouda H., Iino Y., Someya T., Tamagawa Y., Takiyama Y. (2003). Temporal bone histopathological and quantitative analysis of mitochondrial DNA in MELAS. Laryngoscope.

[139] Brown B.G., Zhao X.-Q., Chait A., Fisher L.D., Cheung M.C., Morse J.S., Dowdy A.A., Marino E.K., Bolson E.L., Alaupovic P. (2001). Simvastatin and niacin, antioxidant vitamins, or the combination for the prevention of coronary disease. N. Engl. J. Med..

[140] Sugiyama K., Akai H., Muramatsu K. (1986). Effects of methionine and related compounds on plasma cholesterol level in rats fed a high cholesterol diet. J. Nutr. Sci. Vitaminol..

[141] Ebadi M., Sharma S.K., Wanpen S., Amornpan A. (2004). Coenzyme Q10 inhibits mitochondrial complex-1 down-regulation and nuclear factor-kappa B activation. J. Cell. Mol. Med..

[142] Youn H.-S., Lim H.J., Choi Y.J., Lee J.Y., Lee M.-Y., Ryu J.-H. (2008). Selenium suppresses the activation of transcription factor NF-κB and IRF3 induced by TLR3 or TLR4 agonists. Int. Immunopharmacol..

[143] Segain J.P., De La Blétiere D.R., Bourreille A., Leray V., Gervois N., Rosales C., Ferrier L., Bonnet C., Blottiere H.M., Galmiche J.P. (2000). Butyrate inhibits inflammatory responses through NFκB inhibition: Implications for Crohn’s disease. Gut.

[144] Lührs H., Gerke T., Müller J.G., Melcher R., Schauber J., Boxberger F., Scheppach W., Menzel T. (2002). Butyrate inhibits NF-κB activation in lamina propria macrophages of patients with ulcerative colitis. Scand. J. Gastroenterol..

[145] Hoshino T., Tabuchi K., Nishimura B., Tanaka S., Nakayama M., Ishii T., Warabi E., Yanagawa T., Shimizu R., Yamamoto M. (2011). Protective role of Nrf2 in age-related hearing loss and gentamicin ototoxicity. Biochem. Biophys. Res. Commun..

[146] Honkura Y., Matsuo H., Murakami S., Sakiyama M., Mizutari K., Shiotani A., Yamamoto M., Morita I., Shinomiya N., Kawase T. (2016). NRF2 is a key target for prevention of noise-induced hearing loss by reducing oxidative damage of cochlea. Sci. Rep..

[147] Yang H., Zhu Y., Ye Y., Guan J., Min X., Xiong H. (2022). Nitric oxide protects against cochlear hair cell damage and noise-induced hearing loss through glucose metabolic reprogramming. Free Radic. Biol. Med..

[148] Heinrich U.R., Schmidtmann I., Meuser R., Ernst B.P., Wünsch D., Siemer S., Gribko A., Stauber R.H., Strieth S. (2019). Early Alterations of Endothelial Nitric Oxide Synthase Expression Patterns in the Guinea Pig Cochlea After Noise Exposure. J. Histochem. Cytochem. Off. J. Histochem. Soc..

[149] Rousset F., Nacher-Soler G., Coelho M., Ilmjarv S., Kokje V.B.C., Marteyn A., Cambet Y., Perny M., Roccio M., Jaquet V. (2020). Redox activation of excitatory pathways in auditory neurons as mechanism of age-related hearing loss. Redox Biol..

[150] Ashoori M., Saedisomeolia A. (2014). Riboflavin (vitamin B2) and oxidative stress: A review. Br. J. Nutr..

[151] Chen X., Touyz R.M., Park J.B., Schiffrin E.L. (2001). Antioxidant effects of vitamins C and E are associated with altered activation of vascular NADPH oxidase and superoxide dismutase in stroke-prone SHR. Hypertension.

[152] Joshi R., Adhikari S., Patro B.S., Chattopadhyay S., Mukherjee T. (2001). Free radical scavenging behavior of folic acid: Evidence for possible antioxidant activity. Free Radic. Biol. Med..

[153] Nakagawa K., Ninomiya M., Okubo T., Aoi N., Juneja L.R., Kim M., Yamanaka K., Miyazawa T. (1999). Tea catechin supplementation increases antioxidant capacity and prevents phospholipid hydroperoxidation in plasma of humans. J. Agric. Food Chem..

[154] Papucci L., Schiavone N., Witort E., Donnini M., Lapucci A., Tempestini A., Formigli L., Zecchi-Orlandini S., Orlandini G., Carella G. (2003). Coenzyme q10 prevents apoptosis by inhibiting mitochondrial depolarization independently of its free radical scavenging property. J. Biol. Chem..

[155] Gillis J.C., Benfield P., McTavish D. (1994). Idebenone. Drugs Aging.

[156] Marcocci L., Packer L., Droy-Lefaix M.-T., Sekaki A., Gardès-Albert M. (1994). Antioxidant action of Ginkgo biloba extract EGb 761. Methods in Enzymology.

[157] Packer L., Witt E.H., Tritschler H.J. (1995). Alpha-lipoic acid as a biological antioxidant. Free Radic. Biol. Med..

[158] Turan M., Ciğer E., Arslanoğlu S., Börekci H., Önal K. (2017). Could edaravone prevent gentamicin ototoxicity? An experimental study. Hum. Exp. Toxicol..

[159] Feldman L., Efrati S., Eviatar E., Abramsohn R., Yarovoy I., Gersch E., Averbukh Z., Weissgarten J. (2007). Gentamicin-induced ototoxicity in hemodialysis patients is ameliorated by N-acetylcysteine. Kidney Int..

[160] Takumida M., Popa R., Anniko M. (1999). Free radicals in the guinea pig inner ear following gentamicin exposure. ORL.

[161] Fetoni A.R., Eramo S.L.M., Rolesi R., Troiani D., Paludetti G. (2012). Antioxidant treatment with coenzyme Q-ter in prevention of gentamycin ototoxicity in an animal model. Acta Otorhinolaryngol. Ital..

[162] Sha S.-H., Schacht J. (2000). Antioxidants attenuate gentamicin-induced free radical formation in vitro and ototoxicity in vivo: D-methionine is a potential protectant. Hear. Res..

[163] Kalinec G.M., Fernandez-Zapico M.E., Urrutia R., Esteban-Cruciani N., Chen S., Kalinec F. (2005). Pivotal role of Harakiri in the induction and prevention of gentamicin-induced hearing loss. Proc. Natl. Acad. Sci. USA.

[164] Lynch E.D., Gu R., Pierce C., Kil J. (2005). Reduction of acute cisplatin ototoxicity and nephrotoxicity in rats by oral administration of allopurinol and ebselen. Hear. Res..

[165] Rybak L.P., Husain K., Morris C., Whitworth C., Somani S. (2000). Effect of protective agents against cisplatin ototoxicity. Otol. Neurotol..

[166] Kim J., Cho H.-J., Sagong B., Kim S.-J., Lee J.-T., So H.-S., Lee I.-K., Kim U.-K., Lee K.-Y., Choo Y.-S. (2014). Alpha-lipoic acid protects against cisplatin-induced ototoxicity via the regulation of MAPKs and proinflammatory cytokines. Biochem. Biophys. Res. Commun..

[167] Campbell K.C.M., Rybak L.P., Meech R.P., Hughes L. (1996). D-methionine provides excellent protection from cisplatin ototoxicity in the rat. Hear. Res..

[168] Dickey D.T., Muldoon L.L., Kraemer D.F., Neuwelt E.A. (2004). Protection against cisplatin-induced ototoxicity by N-acetylcysteine in a rat model. Hear. Res..

[169] Riga M.G., Chelis L., Kakolyris S., Papadopoulos S., Stathakidou S., Chamalidou E., Xenidis N., Amarantidis K., Dimopoulos P., Danielides V. (2013). Transtympanic injections of N-acetylcysteine for the prevention of cisplatin-induced ototoxicity: A feasible method with promising efficacy. Am. J. Clin. Oncol..

[170] Takumida M., Anniko M. (2009). Radical scavengers for elderly patients with age-related hearing loss. Acta Otolaryngol..

[171] Salami A., Mora R., Dellepiane M., Manini G., Santomauro V., Barettini L., Guastini L. (2010). Water-soluble coenzyme Q10 formulation (Q-TER^®^) in the treatment of presbycusis. Acta Otolaryngol..

[172] Ohinata Y., Yamasoba T., Schacht J., Miller J.M. (2000). Glutathione limits noise-induced hearing loss. Hear. Res..

[173] Yamasoba T., Nuttall A.L., Harris C., Raphael Y., Miller J.M. (1998). Role of glutathione in protection against noise-induced hearing loss. Brain Res..

[174] Fetoni A.R., Piacentini R., Fiorita A., Paludetti G., Troiani D. (2009). Water-soluble Coenzyme Q10 formulation (Q-ter) promotes outer hair cell survival in a guinea pig model of noise induced hearing loss (NIHL). Brain Res..

[175] Campbell K.C.M., Meech R.P., Klemens J.J., Gerberi M.T., Dyrstad S.S.W., Larsen D.L., Mitchell D.L., El-Azizi M., Verhulst S.J., Hughes L.F. (2007). Prevention of noise-and drug-induced hearing loss with D-methionine. Hear. Res..

[176] Seidman M., Babu S., Tang W., Naem E., Quirk W.S. (2003). Effects of resveratrol on acoustic trauma. Otolaryngol. Neck Surg..

[177] Fetoni A.R., Mancuso C., Eramo S.L.M., Ralli M., Piacentini R., Barone E., Paludetti G., Troiani D. (2010). In vivo protective effect of ferulic acid against noise-induced hearing loss in the guinea-pig. Neuroscience.

[178] Le Prell C.G., Yamashita D., Minami S.B., Yamasoba T., Miller J.M. (2007). Mechanisms of noise-induced hearing loss indicate multiple methods of prevention. Hear. Res..

[179] McFadden S.L., Woo J.M., Michalak N., Ding D. (2005). Dietary vitamin C supplementation reduces noise-induced hearing loss in guinea pigs. Hear. Res..

[180] Yamasoba T., Pourbakht A., Sakamoto T., Suzuki M. (2005). Ebselen prevents noise-induced excitotoxicity and temporary threshold shift. Neurosci. Lett..

[181] Pourbakht A., Yamasoba T. (2003). Ebselen attenuates cochlear damage caused by acoustic trauma. Hear. Res..

[182] Wu F., Xiong H., Sha S. (2020). Noise-induced loss of sensory hair cells is mediated by ROS/AMPKα pathway. Redox Biol..

[183] Davis R.R., Custer D.A., Krieg E., Alagramam K. (2010). N-Acetyl L-Cysteine does not protect mouse ears from the effects of noise. J. Occup. Med. Toxicol..

[184] Kramer S., Dreisbach L., Lockwood J., Baldwin K., Kopke R., Scranton S., O’Leary M. (2006). Efficacy of the antioxidant N-acetylcysteine (NAC) in protecting ears exposed to loud music. J. Am. Acad. Audiol..

[185] Etminan M., Gill S.S., Samii A. (2005). Intake of vitamin E, vitamin C, and carotenoids and the risk of Parkinson’s disease: A meta-analysis. Lancet Neurol..

[186] Lima L.A.R., Lopes M.J.P., Costa R.O., Lima F.A.V., Neves K.R.T., Calou I.B.F., Andrade G.M., Viana G.S.B. (2018). Vitamin D protects dopaminergic neurons against neuroinflammation and oxidative stress in hemiparkinsonian rats. J. Neuroinflamm..

[187] Knekt P., Kilkkinen A., Rissanen H., Marniemi J., Sääksjärvi K., Heliövaara M. (2010). Serum vitamin D and the risk of Parkinson disease. Arch. Neurol..

[188] Suzuki M., Yoshioka M., Hashimoto M., Murakami M., Noya M., Takahashi D., Urashima M. (2013). Randomized, double-blind, placebo-controlled trial of vitamin D supplementation in Parkinson disease. Am. J. Clin. Nutr..

[189] Fullard M.E., Xie S.X., Marek K., Stern M., Jennings D., Siderowf A., Willis A.W., Chen-Plotkin A.S. (2017). Vitamin D in the Parkinson associated risk syndrome (PARS) study. Mov. Disord..

[190] Nakashima H., Ishihara T., Yokota O., Terada S., Trojanowski J.Q., Lee V.M.-Y., Kuroda S. (2004). Effects of α-tocopherol on an animal model of tauopathies. Free Radic. Biol. Med..

[191] Masaki K.H., Losonczy K.G., Izmirlian G., Foley D.J., Ross G.W., Petrovitch H., Havlik R., White L.R. (2000). Association of vitamin E and C supplement use with cognitive function and dementia in elderly men. Neurology.

[192] Maison S.F., Liu X.-P., Eatock R.A., Sibley D.R., Grandy D.K., Liberman M.C. (2012). Dopaminergic Signaling in the Cochlea: Receptor Expression Patterns and Deletion Phenotypes. J. Neurosci..

[193] Du X., West M.B., Cai Q., Cheng W., Ewert D.L., Li W., Floyd R.A., Kopke R.D. (2017). Antioxidants reduce neurodegeneration and accumulation of pathologic Tau proteins in the auditory system after blast exposure. Free Radic. Biol. Med..

[194] Su C., Yan L., Lewith G., Liu J. (2013). Chinese herbal medicine for idiopathic sudden sensorineural hearing loss: A systematic review of randomised clinical trials. Clin. Otolaryngol..

[195] Kaya H., Koç A.K., Sayın İ., Güneş S., Altıntaş A., Yeğin Y., Kayhan F.T. (2015). Vitamins A, C, and E and selenium in the treatment of idiopathic sudden sensorineural hearing loss. Eur. Arch. Oto-Rhino-Laryngol..

[196] Curhan S.G., Stankovic K.M., Eavey R.D., Wang M., Stampfer M.J., Curhan G.C. (2015). Carotenoids, vitamin A, vitamin C, vitamin E, and folate and risk of self-reported hearing loss in women. Am. J. Clin. Nutr..

[197] Shargorodsky J., Curhan S.G., Eavey R., Curhan G.C. (2010). A prospective study of vitamin intake and the risk of hearing loss in men. Otolaryngol. Neck Surg..

[198] Gopinath B., Flood V.M., McMahon C.M., Burlutsky G., Spankovich C., Hood L.J., Mitchell P. (2011). Dietary antioxidant intake is associated with the prevalence but not incidence of age-related hearing loss. J. Nutr. Health Aging.

[199] Aruoma O.I., Hayashi Y., Marotta F., Mantello P., Rachmilewitz E., Montagnier L. (2010). Applications and bioefficacy of the functional food supplement fermented papaya preparation. Toxicology.

[200] Fardet A., Rock E. (2018). In vitro and in vivo antioxidant potential of milks, yoghurts, fermented milks and cheeses: A narrative review of evidence. Nutr. Res. Rev..

[201] Da-Costa-Rocha I., Bonnlaender B., Sievers H., Pischel I., Heinrich M. (2014). Hibiscus sabdariffa L.–A phytochemical and pharmacological review. Food Chem..

[202] Nieto G., Ros G., Castillo J. (2018). Antioxidant and antimicrobial properties of rosemary (*Rosmarinus officinalis* L.): A review. Medicines.

[203] Cabrera C., Artacho R., Giménez R. (2006). Beneficial effects of green tea—A review. J. Am. Coll. Nutr..

[204] Gupta S., Abu-Ghannam N. (2011). Bioactive potential and possible health effects of edible brown seaweeds. Trends Food Sci. Technol..

[205] Dimidi E., Cox S.R., Rossi M., Whelan K. (2019). Fermented Foods: Definitions and Characteristics, Impact on the Gut Microbiota and Effects on Gastrointestinal Health and Disease. Nutrients.

[206] Zhang J., Mori A., Chen Q., Zhao B. (2006). Fermented papaya preparation attenuates β-amyloid precursor protein: β-amyloid–mediated copper neurotoxicity in β-amyloid precursor protein and β-amyloid precursor protein Swedish mutation overexpressing SH-SY5Y cells. Neuroscience.

[207] Logozzi M., Di Raimo R., Mizzoni D., Andreotti M., Spada M., Macchia D., Fais S. (2020). Beneficial Effects of Fermented Papaya Preparation (FPP^®^) Supplementation on Redox Balance and Aging in a Mouse Model. Antioxidants.

[208] Fardet A., Boirie Y. (2014). Associations between food and beverage groups and major diet-related chronic diseases: An exhaustive review of pooled/meta-analyses and systematic reviews. Nutr. Rev..

[209] Ismail A., Ikram E.H.K., Nazri H.S.M. (2008). Roselle (*Hibiscus sabdariffa* L.) seeds-nutritional composition, protein quality and health benefits. Food.

[210] Olaleye M.T., Rocha B.T.J. (2008). Acetaminophen-induced liver damage in mice: Effects of some medicinal plants on the oxidative defense system. Exp. Toxicol. Pathol..

[211] Mohd-Esa N., Hern F.S., Ismail A., Yee C.L. (2010). Antioxidant activity in different parts of roselle (*Hibiscus sabdariffa* L.) extracts and potential exploitation of the seeds. Food Chem..

[212] Frank T., Netzel G., Kammerer D.R., Carle R., Kler A., Kriesl E., Bitsch I., Bitsch R., Netzel M. (2012). Consumption of Hibiscus sabdariffa L. aqueous extract and its impact on systemic antioxidant potential in healthy subjects. J. Sci. Food Agric..

[213] Wu J.W., Lee M.-H., Ho C.-T., Chang S.S. (1982). Elucidation of the chemical structures of natural antioxidants isolated from rosemary. J. Am. Oil Chem. Soc..

[214] Habtemariam S. (2016). The Therapeutic Potential of Rosemary (*Rosmarinus officinalis*) Diterpenes for Alzheimer’s Disease. Evid. Based. Complement. Alternat. Med..

[215] Perry N.S.L., Menzies R., Hodgson F., Wedgewood P., Howes M.-J.R., Brooker H.J., Wesnes K.A., Perry E.K. (2018). A randomised double-blind placebo-controlled pilot trial of a combined extract of sage, rosemary and melissa, traditional herbal medicines, on the enhancement of memory in normal healthy subjects, including influence of age. Phytomedicine.

[216] Li Y.H., Jiang P.S., Su Y.W., Liu Y.M. (2017). Role of green tea polyphenols in noise-induced hearing loss. Chinese J. Ind. Hyg. Occup. Dis..

[217] Gu L.-T., Yang J., Su S.-Z., Liu W.-W., Shi Z.-G., Wang Q.-R. (2015). Green tea polyphenols protects cochlear hair cells from ototoxicity by inhibiting Notch signalling. Neurochem. Res..

[218] Heo S.-J., Park E.-J., Lee K.-W., Jeon Y.-J. (2005). Antioxidant activities of enzymatic extracts from brown seaweeds. Bioresour. Technol..

[219] Chang M.Y., Byon S.-H., Shin H.-C., Han S.E., Kim J.Y., Byun J.Y., Lee J.D., Park M.K. (2016). Protective effects of the seaweed phlorotannin polyphenolic compound dieckol on gentamicin-induced damage in auditory hair cells. Int. J. Pediatr. Otorhinolaryngol..

[220] Zhang L., Du Z., He L., Liang W., Liu K., Gong S. (2022). ROS-Induced Oxidative Damage and Mitochondrial Dysfunction Mediated by Inhibition of SIRT3 in Cultured Cochlear Cells. Neural Plast..

[221] Gao Y., Kamogashira T., Fujimoto C., Iwasaki S., Yamasoba T. (2022). Pyrroloquinoline quinone (PQQ) protects mitochondrial function of HEI-OC1 cells under premature senescence. npj Aging.

[222] Ki D.-W., Kim D.-W., Song J.-G., Woo E.-E., Choi D.-C., Lee I.-K., Yun B.-S. (2022). New antioxidants from the culture broth of Coprinopsis echinospora. J. Antibiot..

[223] Lutchmanen Kolanthan V., Brown A., Soobramaney V., Philibert E.G., Francois Newton V., Hosenally M., Sokeechand B.N., Petkar G., Moga A., Andres P. (2022). Clinical Evaluation of Indian Sandalwood Oil and Its Protective Effect on the Skin against the Detrimental Effect of Exposome. Cosmetics.

[224] Tadokoro K., Morihara R., Ohta Y., Hishikawa N., Kawano S., Sasaki R., Matsumoto N., Nomura E., Nakano Y., Takahashi Y. (2019). Clinical Benefits of Antioxidative Supplement Twendee X for Mild Cognitive Impairment: A Multicenter, Randomized, Double-Blind, and Placebo-Controlled Prospective Interventional Study. J. Alzheimer’s Dis..

[225] Miyata H., Toyoda Y., Takada T., Hiragi T., Kubota Y., Shigesawa R., Koyama R., Ikegaya Y., Suzuki H. (2022). Identification of an exporter that regulates vitamin C supply from blood to the brain. iScience.

[226] Francois-Newton V., Brown A., Andres P., Mandary M.B., Weyers C., Latouche-Veerapen M., Hettiarachchi D. (2021). Antioxidant and anti-aging potential of Indian sandalwood oil against environmental stressors in vitro and ex vivo. Cosmetics.

[227] Liu X., Yamashita T., Shang J., Shi X., Morihara R., Huang Y., Sato K., Takemoto M., Hishikawa N., Ohta Y. (2019). Twendee X Ameliorates Phosphorylated Tau, α-Synuclein and Neurovascular Dysfunction in Alzheimer’s Disease Transgenic Mice With Chronic Cerebral Hypoperfusion. J. Stroke Cerebrovasc. Dis..

[228] Hu X., Yamashita T., Yu H., Bian Z., Hu X., Feng T., Tadokoro K., Morihara R., Abe K. (2021). Neuroprotective and Therapeutic Effects of Tocovid and Twendee-X on Aβ Oligomer-Induced Damage in the SH-SY5Y Cell Line. Neurodegener. Dis..

[229] Lin C.-Y., Wu J.-L., Shih T.-S., Tsai P.-J., Sun Y.-M., Ma M.-C., Guo Y.L. (2010). N-Acetyl-cysteine against noise-induced temporary threshold shift in male workers. Hear. Res..

[230] Brigelius-Flohé R., Traber M.G. (1999). Vitamin E: Function and metabolism. FASEB J..

[231] Oo S.M., Oo H.K., Takayama H., Ishii K., Takeshita Y., Goto H., Nakano Y., Kohno S., Takahashi C., Nakamura H. (2022). Selenoprotein P-mediated reductive stress impairs cold-induced thermogenesis in brown fat. Cell Rep..

